# The 4Fs of cotton: genome editing of cotton for fiber, food, feed, and fuel to achieve zero hunger

**DOI:** 10.3389/fgeed.2024.1401088

**Published:** 2024-09-12

**Authors:** Muhammad Sulyman Saleem, Sultan Habibullah Khan, Aftab Ahmad, Iqrar Ahmad Rana, Zunaira Afzal Naveed, Azeem Iqbal Khan

**Affiliations:** ^1^ Centre of Agricultural Biochemistry and Biotechnology (CABB), University of Agriculture Faisalabad, Faisalabad, Pakistan; ^2^ Center for Advanced Studies in Agriculture and Food Security (CAS-AFS), University of Agriculture Faisalabad, Faisalabad, Pakistan; ^3^ Department of Biochemistry, University of Agriculture Faisalabad, Faisalabad, Pakistan; ^4^ Department of Plant Breeding and Genetics, University of Agriculture Faisalabad, Faisalabad, Pakistan

**Keywords:** cotton, fiber, food, feed, fuel, food security, genome editing, CRISPR/Cas systems

## Abstract

Cotton is globally known for its high-priority cellulose-rich natural fiber. In addition to providing fiber for the textile industry, it is an important source material for edible oil, livestock feed, and fuel products. Global warming and the growing population are the major challenges to the world’s agriculture and the potential risks to food security. In this context, improving output traits in cotton is necessary to achieve sustainable cotton production. During the last few years, high throughput omics techniques have aided in identifying crucial genes associated with traits of cotton fiber, seed, and plant architecture which could be targeted with more precision and efficiency through the CIRPSR/Cas-mediated genome editing technique. The various CRISPR/Cas systems such as CRISPR/Cas9, CRISPR/nCas9, and CRISPR/Cas12a have been employed to edit cotton genes associated with a wide range of traits including fiber length, flowering, leaf colour, rooting, seed oil, plant architecture, gossypol content, somatic embryogenesis, and biotic and abiotic stresses tolerance, highlighting its effectiveness in editing the cotton genome. Thus, CRISPR/Cas-mediated genome editing has emerged as a technique of choice to tailor crop phenotypes for better yield potential and environmental resilience. The review covers a comprehensive analysis of cotton phenotypic traits and their improvement with the help of the latest genome editing tools to improve fiber, food, feed, and fuel-associated genes of cotton to ensure food security.

## 1 Economic importance of cotton in global food security

Cotton, the leading fiber-yielding crop, is commercially cultivated in approximately fifty countries across mild and hot climatic regions. Its cultivation serves to fulfill the demands of different industrial sectors worldwide. Because of the favorable climate conditions required for its natural growth, cotton is grown in various countries, including the USA, South Asia, East Asia, Central Asia, the Middle East, Australia, and Europe (Smith, 1999). The globally recognized dicotyledonous genus *Gossypium* includes approximately fifty species, but only four species; *Gossypium arboreum* L., *Gossypium herbaceum* L., *Gossypium hirsutum* L., and *Gossypium barbadense* L. are commercially cultivated worldwide (Wendel and Grover, 2015). Tetraploid species (*G. hirsutum* L. and *G. barbadense* L.) dominate cultivation in over 80% of global cotton regions, while diploid species (*G. arboreum* L. and *G. herbaceum* L.) are limited to Asia and the Middle East.

**FIGURE 1:**
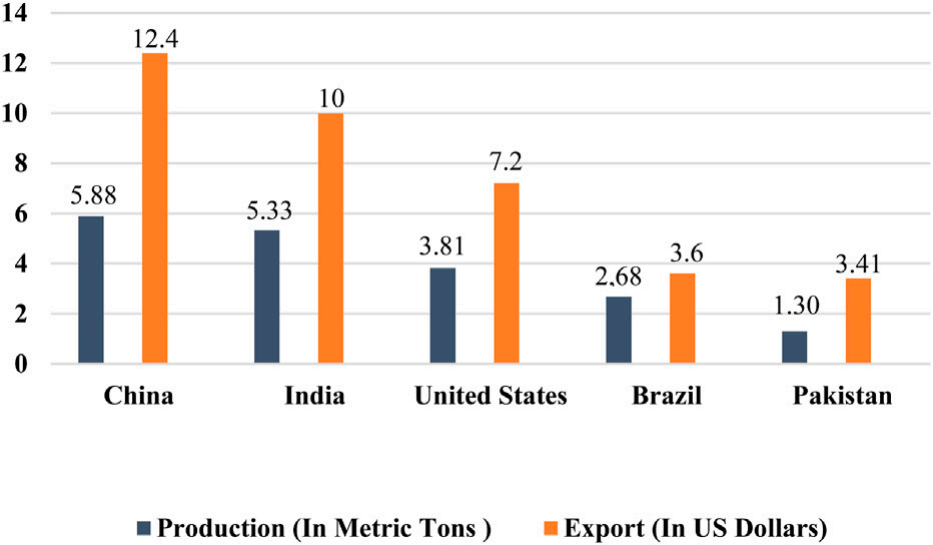
Cotton production and export of the world’s major cotton-growing countries. China is the top producer (5.88 metric tons) and exporter (US $12.4 billion) of cotton among the major cotton-producing countries around the globe (Statista, 2022b).

The agriculture sector plays a pivotal role in the economies of several countries worldwide, with cotton commonly referred to as “white gold” because of its significant profitability for cotton-producing countries. The textile sector exerts a substantial economic impact and accounts for nearly 4% of the global market share, with the cotton part accounting for a share of 30.2% in the worldwide textile market (GITNUX, 2024). This sector is experiencing continuous expansion, mainly driven by the utilization of natural fibers supplied by the cotton crop. Cotton is a vital revenue source for nearly one billion individuals, including 250 million laborers within the cotton industry and 100 million farmers. Notably, about 90% of these farmers cultivate cotton on agricultural land of less than 2 hectares, primarily situated in developing countries (Aslam et al., 2020). Annually, around 25 million tons of cotton are produced globally, covering approximately 14 million acres of cotton-planted areas. The estimated value of this cotton production amounts to around 12 billion dollars (Ahmad and Hasanuzzaman, 2020). Furthermore, in 2021, the global export of cotton reached a total of US $60.4 billion from all countries (Workman, 2022). In 2021, the top five cotton-producing countries were China, India, the United States, Brazil, and Pakistan according to Statista (2022b) (Figure 1). China, being a country with a massive population, holds the distinction of being the world’s largest producer, consumer, and exporter of cotton. It produced approximately 5.88 million metric tons of cotton annually and exported cotton worth approximately US $12.4 billion. Cotton cultivation and processing in China engage an estimated 300 million people, highlighting the substantial workforce dedicated to the industry. India, on the other hand, is the second-largest cotton producer globally, with an annual production of approximately 5.33 million metric tons. India’s cotton exports are valued at approximately US $10 billion (Statista, 2022a). The USA ranks as the third-largest cotton producer globally, with an annual cotton production of about 3.81 million metric tons. The country’s cotton fiber exports amount to a value of US $7.2 billion each year. The overall worth of cotton fiber cultivated annually in the US is estimated to be around US $6 billion, including an additional US $500 million generated from cottonseed oil and its by-products (National Cotton Council of America, 2024). Brazil holds the position of the fourth-largest cotton producer globally, with an annual production of around 2.68 million metric tons. The export value of Brazilian cotton amounts to around US $3.6 billion (Statista, 2022b). Pakistan is indeed recognized as the fourth-largest cotton producer globally, with an annual production of approximately 1.30 million metric tons and export earnings of around US $3.41 billion from cotton exports. Cotton is a major cash crop in Pakistan and plays a vital role in providing essential income to the country’s population. The economy significantly relies on it and the cotton-textile sectors. Pakistan’s cotton textile industry, as the largest exporter of cotton yarn, contributes to 11% of the country’s GDP and accounts for 60% of export income (Economic Survey of Pakistan, 2017). The progress made in the agriculture sector has shown to have a greater impact on enhancing the capability of countries to develop their economies and agricultural markets compared to development in other sectors. Cotton production generates income for nearly 250 million individuals worldwide and employs approximately 7% of the total labor force in developing countries. Hence, the agriculture sector plays a crucial role in accelerating economic growth towards achieving high-income status, while also addressing food insecurity and improving nutrition in developing countries.

In the present era, the global agricultural sector is confronted with two major challenges. Firstly, the world population is continuously growing and is predicted to surpass 9 billion by 2050 (United Nations, 2017). This population expansion poses significant concern in meeting the escalating demand for essential resources such as fiber, food, fuel, and feed (Silva, 2018; Parihar et al., 2022). Secondly, global warming poses a prominent threat to agricultural yields worldwide. Predictions indicate that the world’s temperature could rise by 1.5°C by 2030 which has a considerable risk to agricultural productivity and the overall stability of the agricultural sector. Extreme global temperatures contribute to various detrimental effects on agricultural systems, leading to heat stress and exacerbating the intensity of abiotic and biotic stresses. These include the occurrence of severe and persistent droughts, soil salinization, altered rainfall patterns, increased weed growth, and a rise in the population of plant pathogens and insect pests (Arora, 2019). Indeed, the frequent climate changes, accompanied by water resource constraints and various abiotic and biotic stresses, have led to a decline in agronomic crop yield and quality. To address this challenge, it becomes imperative to enhance food production by two to three times and develop climate-resilient agronomic crops. Achieving food security requires increasing the income and productivity of farmers, reducing food prices, and improving overall nutrition. Therefore, it is crucial to prioritize the improvement in the yield and quality of agronomic crops to combat hunger and malnutrition, both in the present and the future.

## 2 The 4Fs of cotton

**FIGURE 2:**
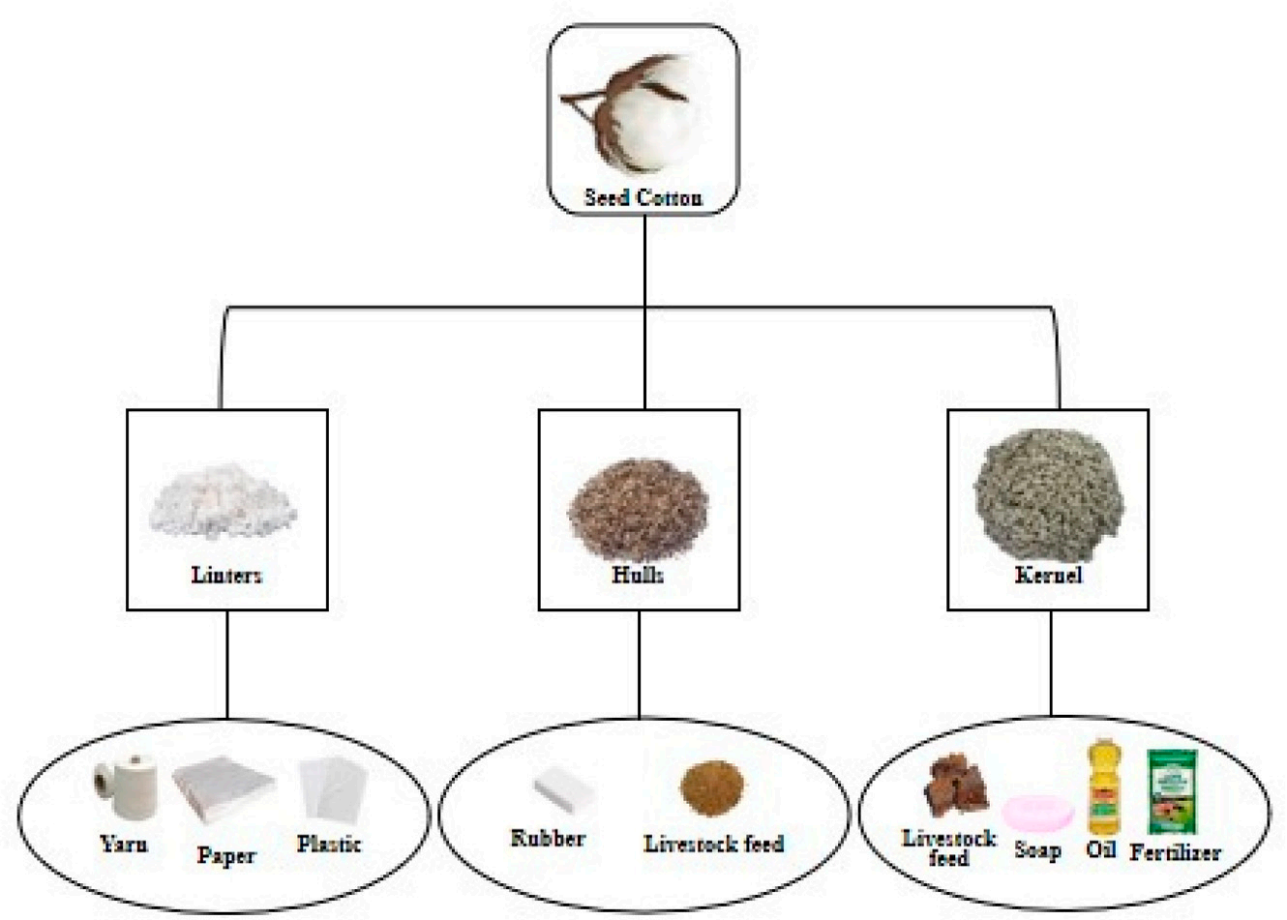
Illustrative overview of consumer products derived from seed cotton. Cotton fiber, cottonseed cake, and cottonseed oil are the main agricultural products, which contribute to cotton’s economic value by offering key materials for various industries.

Cotton stands as a primary agronomic crop cultivated worldwide due to its multifaceted uses in fiber, food, feed, and fuel (4Fs) (Yin and Ding, 2021). It serves as a valuable source of renewable resources, as ginned cottonseed comprises various components, including 16% crude oil and 46% meal in the kernel, along with 27% hull and 8% linters (Chaudry and Guitchounts, 2022). The 4Fs of cotton play a crucial role in enhancing global food security by empowering cotton-growing farmers economically. The influence of cotton production extends to multiple industrial sectors such as textiles, plastics, paper, livestock feed, soap, and oil (Figure 2).

### 2.1 Fiber

Cotton fiber is considered the purest and most vital naturally produced cellulosic fiber. Typically, a cotton fiber comprises approximately 80%–90% cellulose, 4%–6% hemicelluloses, 0.5%–1% waxes, 0%–1.5% proteins, 6%–8% water, and 8% ash, pectin, and fats (Cotton Incorporated, 2020). Because of its remarkable strength, durability, and comfort, cotton fiber is ideally suited for making clothes, housewares, and other textile products. The economic significance of cotton fiber in the global market is substantial, accounting for over 50% of textile products. The cotton fiber quality directly impacts the overall quality of cotton fiber-based garments, underscoring its significance. By 2050, it is predicted that approximately 8 billion people will require clothing made from cotton fiber, leading to a significant increase in the annual demand for cellulosic fiber, from 50 million to 130 million tons. So, the crucial role of cotton will continue to play in fulfilling the global need for natural fiber.

### 2.2 Food

Cotton also serves the edible oil industry, making about 6 million tons of cottonseed oil annually and producing around US $4 billion in revenue (Statista, 2022a). Amid increasing edible oil costs and shortages, cottonseed oil is necessary for ensuring nutritious cooking oil, especially in developing countries. With 21% oil and 23% protein content, cottonseed can address the protein needs of almost a billion people, especially in malnourished regions such as Asia and Africa (Kumar et al., 2021). Post oil extraction, the high-protein meal can be used for flour or snacks, potentially fulfilling the daily protein needs of 575 million people. Globally, cottonseed yields 11 million tons of protein, sufficient for the annual requirements of around 600 million people. Rich in vitamins and minerals, cottonseed is significant for alleviating food crises and increasing nutrition without additional resources.

### 2.3 Feed

Cottonseed meal, a byproduct of oil extraction, is an important and cost-effective protein source for livestock feed, making up 30%–40% of the protein content, and increasing cattle production because of its high phosphorus content (Hernandez, 2016). Cottonseed hulls offer fiber-rich roughage for grain-based diets, even with low energy and digestibility (C H Knight Limited, 2024). Global animal feed production is approximately one billion tons annually, generating around US $400 billion in revenue for the animal feed industry (International Feed Industry Federation, 2024). With increasing global requirements for livestock, fish, and dairy products driven by population growth and enhancing urban incomes, the FAO predicts significant increases in meat (70%), dairy (55%), and aquaculture (90%) production. Cottonseed’s high energy value makes it indispensable in meeting this rising demand.

### 2.4 Fuel

Energy is a fundamental requirement for human survival and economic development, and cotton-derived biofuel provides a significant renewable energy source. Now agricultural biomass accounts for around 15% of the world’s primary energy (Bassam, 2001). In industrial countries, agricultural biomass produces “green electricity” and thermal energy, with crops converted into biofuels in liquid or gaseous forms (Luterbacher and Luterbacher, 2015). Cotton-derived biofuel offers renewable, eco-friendly alternatives to fossil fuels, ensuring future energy security and decreasing reliance on imports. However, the availability of cottonseed oil is restricted based on cotton production and seasonality, affecting biofuel supply consistency. The extraction and conversion process needs significant capital and technological advancements to be economically sustainable (Soomro et al., 2023). Cotton-derived biofuel competes with other biofuels and fossil fuels and faces challenges associated with land and water use, food security, biodiversity, public acceptance, and complex regulations (Soomro et al., 2023). Nonetheless, to overcome these challenges, cotton-derived biofuel holds the potential for sustainable energy.

## 3 Traits and genes associated with 4Fs of cotton

The yield of cotton cultivation encompasses two main components: the seed and the fiber, known as lint. These components have the potential to provide food, feed, fiber, and fuel, commonly referred to as the 4Fs. Understanding and characterizing cotton genes and their associated traits related to the 4Fs is crucial for comprehending the functional genetics of cotton and effectively improving traits associated with yield and quality. The advancements in molecular genetics, transgenic techniques, whole genome sequencing, and bioinformatics have revolutionized crop breeding studies, providing new tools that prioritize gene-based approaches for trait improvement. Therefore, knowledge about the genetic control of agronomical traits is indispensable for enhancing seed and fiber quality and overall yield in cotton. In this section, we discuss previous studies that have explored cotton gene function and genes with significant impacts on cotton yield and quality through upregulation or downregulation of their expression. Manipulating these genes presents an opportunity to improve cotton’s desired output traits.

### 3.1 Fiber-related genes and traits

Cotton fiber is an immensely significant natural textile fiber globally. Each long cotton fiber originates from a single epidermal cell located on the surface of the ovule. Through the process of cell wall thickening, this cell transforms into an extremely elongated and durable dead fiber. The development of the fiber progresses through distinct stages, including initiation, elongation, transitional primary wall remodeling, secondary wall synthesis, and maturation. These stages directly determine the quality properties of cotton fiber. The yield of fiber is influenced by the number of fibers originating from the outer surface of the ovule. Numerous studies have been conducted to identify and characterize genes associated with fiber development and their roles in improving fiber quality. Some genes exhibit preferential expression during the fiber initiation stage, while others show a high level of expression during the fiber elongation stage (Table 1).

**TABLE 1:** A list of genes and traits associated with fiber.

Gene	Specific trait	References
Actin1 (*GhACT1*)	Fiber elongation	Li et al. (2005)
*GhBIN 2*	Fiber elongation	Sun et al. (2023)
Profilin 1 (*GhPFN1*)	Fiber elongation	Wang et al. (2005)
*GhMYB-25* ^a^	Fiber elongation	Machado et al. (2009)
*GhMADS11*	Fiber elongation	Li et al. (2011)
Sucrose synthase (*GhSUSA1*)	Fiber length and strength	Jiang et al. (2012)
Protodermal factor 1 (*GBPDF1*)	Fiber initiation	Deng et al. (2011)
*GhGAI3A*	Fiber initiation	Wang et al. (2012)
Fasciclin-like arabinogalactan (*GhFLA1*)	Fiber initiation and elongation	Huang et al. (2013)
Plasma membrane intrinsic protein (*GhPIP2*)	Fiber elongation	Li et al. (2013)
Teosinte-branched1/cycloidea/pcf (tcp) protein (*GhTCP 14*)	Fiber elongation	Wang et al. (2013)
Proline-rich protein (*GhPRP5*)	Fiber length	Xu et al. (2013)
Vacuolar invertase (*GhVIN1*)	Fiber initiation	Wang et al. (2014b)
*GhHOX3*	Fiber elongation	Shan et al. (2014)
Pagoda 1 (*PAG1*)	Fiber elongation	Yang et al. (2014)
*GhKNL1*	Fiber elongation	Gong et al. (2014)
Phytochrome apoprotein (*GhPHYA1*)	Fiber length	Abdurakhmonov et al. (2014)
Caprice (*GhCPC*)	Fiber initiation and elongation	Liu et al. (2015)
Calmodulin (*GhCAM7*)	Fiber elongation	Cheng et al. (2016)
Jasmonate zim-domain 2 (*GhJAZ2*)	Fiber initiation	Hu et al. (2016)
Β-galactosyltransferase 1 (*GhGALT1*)	Fiber length	Qin et al. (2017)
*GhFSN1*	Fiber strength	Zhang et al. (2017b)
Histone deacetylase (*GhHDA5*)	Fiber initiation	Kumar et al. (2018)
Ubiquitin ligase (*GhHUB2*)	Fiber elongation	Feng et al. (2018)
C2H2-zinc finger	Fiber initiation	Salih et al. (2019)
*GhBHLH18*	Fiber strength and length	Gao et al. (2019a)
*GaHD1*	Fiber initiation	Ding et al. (2020)
Alanine rich protein (*GhALARP*)^a^	Fiber elongation	Zhu et al. (2021a)
Phosphoinositide-specific phospholipase C (*GhPIPLC2D*)	Fiber length	Zhu et al. (2021b)
Transparent testa 2 (*GhTT2-3A*)	Fiber color	Yan et al. (2018)
4-coumarate: coenzyme a ligase (*Gh4CL4*)	Fiber color	Sun et al. (2019)
Chalcone synthases (*GhCHS*)	Fiber color	Gao et al. (2019b)
Anthocyanidin reductases (*GhANR*)	Fiber color	Gao et al. (2019a)
Leucoanthocyanidin reductases (*GhLAR*)	Fiber color	Gao et al. (2019b)
Chalcone isomerase (*GhCHI*)	Fiber color	Lv et al. (2023)
Flavanone 3-hydroxylases (*GhF3H*)	Fiber color	Lv et al. (2023)
Flavonoid 3′, 5′-hydroxylases (*GhF3′5′H*)	Fiber color	Lv et al. (2023)
Dihydroflavonol 4-reductases (*GhDFR*)	Fiber color	Lv et al. (2023)

^a^ CRISPR-edited gene.

The PROTODERMAL FACTOR1 gene (*GbPDF1*) is highly expressed in fiber cells during the fiber initiation stage. Its function is to maintain hydrogen peroxide homeostasis and regulate the biosynthesis of ethylene and pectin. This regulation is achieved through the interaction with a key cis-element called HDZIP2ATATHB2 (Deng et al., 2011). The *GhGAI3a* gene, encoding the DELLA protein, shows high expression at the fiber initiation stage in fiber cells. It functions as a repressor in the gibberellin (GA) signaling pathway, playing a role in regulating fiber development (Wang et al., 2012). The FASCICLIN-LIKE ARABINOGALACTAN protein encoded by the *GhFLA1* gene is involved in both the fiber initiation and elongation processes in cotton. It affects the composition of the ARABINOGALACTAN protein (AGP) and the primary cell wall (Huang et al., 2013). The *GhVIN1* gene encodes VACUOLAR INVERTASE (VIN), and it plays a crucial role in fiber initiation. It modulates the transcription of MYB transcription factors and auxin signaling constituents via VIN-based hexose signaling (Wang X. et al., 2014). The R3-MYB gene *GhCPC* encodes the caprice protein, which negatively regulates fiber initiation and early elongation. It potentially forms a complex with CPC-MYC1-TTG1/4 in cotton (Liu et al., 2015). The JASMONATE ZIM-DOMAIN 2 (JAZ2) protein, encoded by the *GhJAZ2* gene, acts as a primary transcription repressor during fiber initiation. It interacts with the *GhMYB-25* transcription factor to modulate the jasmonic acid (JA) signaling pathway (Hu et al., 2016). The *GhHDA5* gene, encoding histone deacetylase, is specifically expressed during fiber initiation and regulates the expression of fiber initiation-specific genes (Kumar et al., 2018). C_2_H_2_-zinc finger proteins encoded by C_2_H_2_-zinc finger genes play an indispensable role in regulating the fiber initiation and development process in cotton (Salih et al., 2019). The *GaHD1* gene, a HOMEODOMAIN-LEUCINE ZIPPER gene, plays a crucial role in cotton fiber initiation. It regulates the signaling cascade involved in hydrogen peroxide (H_2_O_2_) production and calcium ion (Ca2^+^) flux (Ding et al., 2020).

In a study conducted by Li et al. (2005), it was demonstrated that silencing the ACTIN (*GhACT1*) gene through RNA interference (RNAi) in *G. hirsutum* resulted in inhibited fiber elongation. This inhibition was attributed to the disruption of the actin cytoskeleton in fiber cells during the process of fiber development. Sun and Allen (2005) found that the shaggy-like protein kinase family (Bin 2) genes exert a negative regulatory effect on cotton fiber elongation by modulating brassinosteroid (BR) signaling. According to Wang et al. (2005), the *GhPFN1* gene in cotton was found to play a crucial role in facilitating rapid fiber elongation by triggering actin polymerization. Machado et al. (2009) revealed the significance of *GhMYB-25*, a member of the MYB family transcription factors, in regulating cotton fiber elongation. Li et al. (2011) discovered that a MADS-box protein encoded by the *GhMADS11* gene also exerts control over the elongation process of cotton lint fibers. A study indicated that the SUCROSE SYNTHASE (*GhSusA1*) gene is pivotal in determining cotton fiber length and strength by regulating the cell wall thickness during the secondary cell wall development stage (Jiang et al., 2012). In the study by Li et al. (2013), they identified the preferentially expressed *GhPIP2* genes in cotton fibers, which encode an aquaporin known as plasma membrane intrinsic proteins (PIPs). The suppression of *GhPIP2* gene expression through RNAi significantly inhibited fiber elongation, indicating their crucial role in this stage of cotton fiber development. Wang et al. (2013) discovered that a TEOSINTE-BRANCHED1/CYCLOIDEA/PCF (TCP) transcription factor encoded by the *GhTCP14* gene in upland cotton (*Gossypium hirsutum*) serves as a key regulator in the auxin-mediated development and rapid elongation of fiber cells. It achieves this by interacting with the promoters of *GhAUX*, *GhPIN2*, and *GhIAA3* genes. In a previous study, Xu et al. (2013) reported that the anti-sense suppression of the fiber-specific *GhPRP5* gene, which encodes a proline-rich protein, resulted in increased fiber length in cotton. Shan et al. (2014) demonstrated that *GhHOX3* encoded homeodomain-leucine zipper (HD-ZIP) transcription factor, which interacts with *GhHD1* in the gibberellic acid (GA) signaling pathway, contributing to the regulation of fiber elongation. Yang et al. (2014) identified the PAGODA1 (*PAG1*) gene, which plays a role in brassinosteroid (BR) catabolism and fiber elongation by modulating the levels of endogenous brassinosteroid. Additionally, Gong et al. (2014) found that the KNOX protein encoded by the kNOTTED-LIKE (*GhKNL1*) gene is specifically expressed in growing fiber cells during the secondary cell wall biosynthesis stage in cotton, thereby regulating fiber elongation. According to Abdurakhmonov et al. (2014), RNAi-mediated suppression of the *GhPHYA1* gene, which encodes the phytochrome red photoreceptor, led to a reduction in fiber length in cotton plants. Calcium signaling cascades involved in fiber elongation are regulated by *GhCaM7*, which encodes calmodulin, a calcium sensor, and influences calcium ion (Ca2^+^) influx (Cheng et al., 2016). Qin et al. (2017) reported that the *GhGalT1* gene in cotton, belonging to the CAZy glycosyltransferase 31 family, encodes Β-1,3-GALACTOSYLTRANSFERASE. It negatively regulates fiber length and is involved in the biogenesis of the β-1,3-galactan backbone of type-II arabinogalactan glycans. Zhang J. et al. (2017) found that the *GhFSN1* gene, a member of the NAC transcription factors family in cotton, is specifically expressed during the secondary cell wall development stage. It acts as a positive regulator by activating secondary cell wall-associated genes, thereby modulating secondary cell wall thickening in cotton fibers. The *GhHUB2* gene encodes a ubiquitin ligase that regulates fiber length and secondary cell wall thickness in cotton and promotes the degradation of the *GhKNL1* protein (a repressor) through the ubiquitin-26 S proteasome mechanism (Feng et al., 2018). Gao J. et al. (2019) conducted an expression study on the *GhbHLH18* gene, revealing its specific expression during the fiber elongation stage. They found that *GhbHLH18* negatively modulates secondary cell wall development and fiber elongation by interacting with the lignin-specific *GhPER8* gene. In a study by Zhu L. et al. (2021), it was reported that the *GhAlaRP* (alanine-rich protein) gene plays a crucial role in regulating cotton fiber elongation. *GhAlaRP* interacts with *GhAnnexin* and *GhEXPA* genes, and the RNAi-mediated suppression of *GhAlaRP* resulted in reduced fiber length through co-suppression of *GhAnnexin* and *GhEXPA* gene expression in cotton. Furthermore, Zhu S. et al. (2021) demonstrated that the *GhPIPLC2D* (PHOSPHOINOSITIDE-SPECIFIC PHOSPHOLIPASE C) gene is particularly expressed in fibers during the elongation stage. It acts as a positive regulator in fiber elongation by enhancing ethylene biogenesis. Silencing the *GhPIPLC2D* gene led to shorter lint fibers and reduced biosynthesis of Inositol-1, 4, 5-trisphosphate and ethylene.

Naturally colored cotton fibers, such as brown or green, acquire their non-white coloration through the accumulation of pigments during the fiber development process. This eliminates the need for dyeing processes in fabric manufacturing, resulting in reduced expenses and a more environmentally friendly approach by avoiding the disposal of harmful dye waste (Dutt et al., 2004). Plants produce flavonoids, which comprise the largest class of secondary metabolites, including colored compounds that are synthesized and accumulated in developing cotton fibers, giving rise to their natural pigmentation (Mikhailova et al., 2019). The transcriptional regulator *GhTT2-3A* (TRANSPARENT TESTA 2) is predominantly expressed in brown fibers and plays a crucial role in controlling brown pigmentation by regulating proanthocyanidins (PA) biosynthesis (Yan et al., 2018). Furthermore, the *Gh4CL4* gene, encoding 4-coumarate: Coenzyme A ligase, has been found to contribute to the biosynthesis of green pigments in cotton (Sun et al., 2019). Additionally, several genes involved in flavonoid biosynthesis, such as *GhCHS*, *GhANR*, *GhLAR*, *GhCHI, GhF3H*, *GhF3′5′H*, and *GhDFR*, have been identified and shown to be significantly expressed in developing fibers, thereby contributing to the pigmentation in naturally colored cotton (Gao Z. et al., 2019; Lv et al., 2023).

Hence, the cotton fiber-related traits are controlled by the genes involved in plant growth, development, and defensive hormones such as gibberellic acid, jasmonic acid, brassinosteroid, ethylene, abscisic acid, flavonoid, etc., pathways.

### 3.2 Seed-related genes and traits

Cottonseed is a by-product of cotton, characterized by its unique seed structure and high natural pigment content. It serves as a major source of unsaturated edible oil, free from cholesterol, with a composition consisting of 65%–70% polyunsaturated fatty acids, including 18%–24% oleic acid and 42%–52% linoleic acid. It also contains 26%–35% saturated fatty acids, such as palmitic and stearic acids, making it stand out among other seed oil-producing plants (Liu et al., 2002). The relatively high level of saturated fatty acids, particularly palmitic acid, contributes to the oxidative stability of cottonseed oil, making it a reliable choice for frying at high temperatures compared to oils rich in polyunsaturated fatty acids like linoleic acid and oleic acid. However, it is worth noting that saturated fatty acids are not considered reliable and healthy due to their association with an increase in low-density lipoprotein (LDL) cholesterol levels. Researchers have downregulated two essential fatty acid desaturase genes, namely *GhSAD-1* (STEAROYL-ACYL-CARRIER PROTEIN Δ9-DESATURASE) and *GhFAD2-1* (OLEOYL-PHOSPHATIDYLCHOLINE ω6-DESATURASE), through RNA-mediated gene silencing. This genetic modification aims to enhance the oleic acid content in cottonseed oil (Liu et al., 2002). Additionally, Chen et al. (2020) utilized CRISPR/Cas9 mediated editing to knock out the *GhFAD2* genes, resulting in the production of non-transgenic cotton (*Gossypium hirsutum* L.) with a higher level of oleic acid content. Cottonseed consists of a hull and kernel. The hull is used for fiber or linters, while the kernel contains essential nutritional components such as oil, protein, carbohydrates, vitamins, minerals, lecithin, sterols, and more. Wang N. et al. (2017) conducted a genome-wide association study (GWAS) on the cotton LYSOPHOSPHATIDIC ACID ACYLTRANSFERASE (*LPAAT*) gene family, revealing their association with the biosynthesis of cotton seed oil components like palmitic acid, oleic acid, and triacylglycerol.

In a study conducted by Yao et al. (2018), bio-fortification of cottonseed with Pro-vitamin A was achieved through targeted upregulation of the PHYTOENE SYNTHASE (*GhPSY2D*) gene. This was accomplished by introducing a seed-specific promoter, which effectively enhanced the levels of β-carotene in transgenic cottonseeds. This innovative approach to bio-fortify cotton with pro-vitamin A offers a promising solution to address vitamin A deficiency on a global scale, benefiting populations worldwide.

Cottonseed contains certain anti-nutritional or toxic compounds such as gassypurpurin, gossycaerulin, gossyfulivin, gossyverdurin, and gossypol. Among these, gossypol is a yellow terpenoid pigment that plays a vital role in defending cotton plants against insect predation (Scheffler, 2016). Gossypol is present in higher quantities in raw cottonseed compared to cooked cottonseed, posing challenges in processing and consuming cottonseed as a by-product. It is toxic to non-ruminant animals since it exists in a free state within the seed. However, during cooking, gossypol binds to the free amino or free carboxyl groups of cottonseed protein, forming “bound gossypol.” This reduces the nutritional value of the protein and limits the availability of essential amino acids like lysine (Rathore et al., 2020). The consumption of cottonseed containing gossypol can lead to various chronic effects such as liver damage, reproductive and immune toxicity, reduced iron bioavailability, disturbance in iron utilization, and lysine deficiency (Gadelha et al., 2014). To address this issue, researchers have employed seed tissue-specific RNAi to suppress the expression of the δ-CADINENE SYNTHASE (*CAD1*) gene, which disrupts the gossypol biosynthesis pathway during seed development. This approach has significantly reduced gossypol levels in cottonseed without affecting its levels in other plant tissues where it serves as a defense against pests (Sunilkumar et al., 2006). Gao et al. (2020) reported the *CGP1* gene, which encodes a transcription factor preferentially expressed in the black glands of *Gossypium* spp. These glands play a crucial role in storing large amounts of gossypol and other secondary metabolites, providing defense against insects and pathogens (Gao et al., 2020). By using RNAi and CRISPR tools to suppress the expression of the *CGP1* gene, the researchers successfully created a *CGP1* mutant with reduced gossypol levels and a glandless-like phenotype (Gao et al., 2020). Additionally, they suggested that the *CGP1* transcription factor interacts with *GOPGF* in the nucleus, which is a key transcription factor for black gland formation and gossypol biosynthesis (Gao et al., 2020).

We could increase the nutritional content including polyunsaturated fatty acid, and tocopherol, and decrease or eliminate the gossypol and cyclopropane-free fatty content in cottonseed oil through modern biotechnological tools to enhance or suppress the expression of genes that regulate their biosynthesis in cottonseed (Table 2).

**TABLE 2:** A list of genes and traits associated with seed.

Gene	Specific trait	References
Stearoyl-acyl-carrier protein δ9-desaturase (*GhSAD-1*)	Fatty acid desaturation	Liu et al. (2002)
Oleoyl-phosphatidylcholine ω6-desaturase (*GhFAD2-1*)^a^	Fatty acid desaturation	Liu et al. (2002) Chen et al. (2020)
Δ-cadinene synthase (*CAD1*)	Gossypol biosynthesis	Sunilkumar et al. (2006)
Lysophosphatidic acid acyltransferase (*LPAAT*)	Oil biosynthesis	Wang et al. (2017b)
Phytoene synthase (*GhPSY2D*)	Pro-vitamin A biosynthesis	Yao et al. (2018)
*CGP1*	Gossypol biosynthesis	Gao et al. (2020)

^a^ CRISPR-edited gene.

### 3.3 Plant architecture-related genes and traits

Plant architecture plays a fundamental role in the productivity and management of cotton. The structure of a cotton plant is determined by meristems, which undergo differentiation to form a terminal structure during vegetative growth. The primary meristems give rise to branches (monopodial and sympodial), inflorescence, and flowers, while the secondary meristems contribute to woody growth, and organ-specific meristems shape the final structure of organs.

Roots serve as vital organs in crops, responsible for nutrient and water absorption from the soil, starch storage, physical support, and defense against biotic and abiotic stresses. Lateral root development is regulated by nitric oxide, a molecule synthesized from arginine in the presence of the nitric oxide synthase enzyme. However, increased arginase activity can inhibit the function of nitric oxide synthase due to competition for the same arginine substrate by both enzymes. To overcome this limitation, researchers utilized the CRISPR tool to edit the *GhARG* gene, which encodes ARGINASE in cotton plants. By knocking out the function of the *GhARG* gene, lateral root development was enhanced, resulting in increased crop yield (Wang N. et al., 2017).

Branching plays a crucial role in the regulation of above-ground organs in crops, directly influencing their development and yield. The intricate regulatory network responsible for branching involves phytohormones and transcription factors, serving as a fundamental mechanism for plant survival and occupation of space. Several genes involved in branch regulation have been identified and characterized in cotton, presenting potential targets for genetic manipulation of branch architecture (Table 3). McGarry et al. (2016) proposed that the expression levels of cotton SINGLE FLOWER TRUSS (*GhSFT*)-like and SELF-PRUNING (*GhSP*)-like genes dynamically modulate the development of monopodial and sympodial branches. Mutation of the SELF-PRUNING-like genes resulted in the termination of both branch systems in cotton. The cotton CUP-SHAPED COTYLEDON 2 (*GhCUC2*) genes (Zhan et al., 2021), members of the NAC family, participate in auxin and abscisic acid signaling pathways, while the BRANCHED 1 (*GhBRC1*) gene (Sun et al., 2022) from the TCP family acts as a negative regulator of branch development. In a previous study, suppression of the cotton DEHYDRATION-RESPONSIVE ELEMENT-BINDING (*GhDREB*) gene through virus-induced gene silencing (VIGS) significantly increased plant height, branch angle, and height (Ji et al., 2020). The *GhTIE1* gene, encoding a TCP interactor containing an EAR motif protein 1, also promotes shoot branch development in cotton by suppressing the expression of TCPs such as *GhBRC1*, *GhBRC2*, and *GhTCP13* genes (Diao et al., 2019). Furthermore, Zhao et al. (2017) identified and functionally characterized the *GhMAX2* gene in cotton, which encodes an F-box/LRR family protein and acts as a repressor in shoot lateral branch development.

**TABLE 3:** A list of genes and traits associated with plant architecture.

Gene	Specific trait	References
Flowering promoting factor 1 (*GhFPF1*)	Flowering	Wang et al. (2014b)
Self-pruning (*GhSP*)	Branching	McGarry et al. (2016)
Single flower truss (*GhSFT*)	Branching	McGarry et al. (2016)
Phosphatidylethanolamine-binding protein (*GhPEBP*)^a^	Flowering	Zhang et al. (2016)
Late meristem identity 1 (*GhLMI1*)	Leaf shape	Andres et al. (2016)
More axillary growth 2 (*GhMAX2*)	Branching	Zhao et al. (2017)
Arginase (*GhARG*)^a^	Lateral rooting	Wang et al. (2017b)
Constans/flowering locus T (*GhCOL*)	Flowering	Cai et al. (2017)
*GhHB12*	Flowering	He et al. (2018)
Tcp interactor containing ear motif protein 1 (*GhTIE1*)	Branching	Diao et al. (2019)
Α-amylase inhibitor 6 (*GhAA16*)	Flowering	Qanmber et al. (2019)
Dehydration-responsive element-binding (*GhDREB1B*)	Branch height, length, and angle	Ji et al. (2020)
*GhCAL* ^a^	Flowering	Cheng et al. (2020)
Auxin response factor (*GhARF16-1*)	Leaf shape	He et al. (2021)
Apetala1 (*GhAP1*)	Flowering	Cheng et al. (2021)
Cup-shaped cotyledon 2 (*GhCUC2*)	Branching	Zhan et al. (2021)
Branched 1 (*GhBRC1*)	Branching	Sun et al. (2022)

^a^ CRISPR-edited gene.

Leaves serve as the primary site for photosynthesis, making leaf shape a significant trait that influences crop productivity. In cotton, the leaf shape is regulated by various factors. One such factor is an HD-Zip transcription factor encoded by the LATE MERISTEM IDENTITY1 (*GhLMI1*)-like gene, as demonstrated by Andres et al. (2016). This transcription factor plays a role in determining the leaf shape in cotton. Additionally, He et al. (2021) proposed that the cotton AUXIN-RESPONSIVE FACTOR (*GhARF16-1*) specifically binds to the upstream region of *GhKNOX2-1*, another gene involved in leaf development. This binding interaction leads to the modulation of leaf shape by inducing the expression of *GhKNOX2-1*. Overexpression of *GhARF16-1* and *GhKNOX2-1* resulted in increased serrations in cotton leaves.

Crop plants, including cotton, undergo a critical stage transition from vegetative to reproductive development, which significantly impacts early maturity and flowering time (FTi). Early maturity is crucial for lint fiber quality, yield, and mechanical harvesting of cotton. In recent years, several transcriptomic studies have been conducted to identify and characterize genes associated with flowering that regulate flowering time and floral organs in cotton (Table 3). Wang X. et al. (2014) proposed that the FLOWERING PROMOTING FACTOR 1 (*GhFPF1*) gene plays a role in controlling flowering time in cotton. The PHOSPHATIDYLETHANOLAMINE-BINDING PROTEIN (*GhPEBP*)-like gene family also plays a critical role in modulating flowering time and photoperiod responses in cotton (Zhang et al., 2016). In contrast to *GhPEPBP2*, CONSTANS/FLOWERING LOCUS T (*GhFT*) (Cai et al., 2017) binds with FD-like bZIP (*GhFD*) to rescue the late-flowering phenotype in photoperiod-sensitive and wild cotton (Zhang et al., 2016). He et al. (2018) reported that the cotton *GHB12* gene, a member of the HD-ZIP I-class transcription factor family, is preferentially expressed in axillary buds. It negatively regulates flowering by binding to *GhSPL10/13*, suppressing the expression of *GHSOC1, GHFT*, and *GhFUL* genes, resulting in delayed flowering under long-day conditions. The α-AMYLASE INHIBITOR (*GhAAI66*) gene, specifically expressed in floral tissue, promotes early flowering by integrating various floral signaling mechanisms, as shown by its RNAi-mediated silencing in cotton (Qanmber et al., 2019). Two genes of the MADS-box family, *GhCAL* (Cheng et al., 2020) and APETALA1 (*GhAP1*) (Cheng et al., 2021), play a key role in positively regulating flowering time (FTi) and the development of floral organs in cotton. *GhLFY* negatively regulates the expression of *GhAP1* in the regulatory mechanisms (Cheng et al., 2021), and *GhAP1* interacts with *GhSOC1* for the transcriptional activation of multiple flowering-associated genes (Wang C. et al., 2023).

Hence, these reported studies help provide us with knowledge about potential genes for improving cotton plant architecture as well as crop yield through advanced gene editing approaches.

## 4 Conventional breeding approaches

Plant breeding, a crucial science, involves intentionally introducing advantageous and inheritable changes in plants, making it one of the most pivotal approaches to improving crops. The significant impact of the Green Revolution, which has effectively reduced hunger and poverty for millions of people (Pingali, 2012), exemplifies the importance of this field. Selective breeding has played a prominent role in producing the majority of the currently cultivated cotton genotypes, leading to substantial improvements in cotton quality and yield. In this section, we provide a concise overview of various conventional breeding approaches employed for cotton crop improvement along with their limitations.

### 4.1 Selective breeding

**FIGURE 3:**
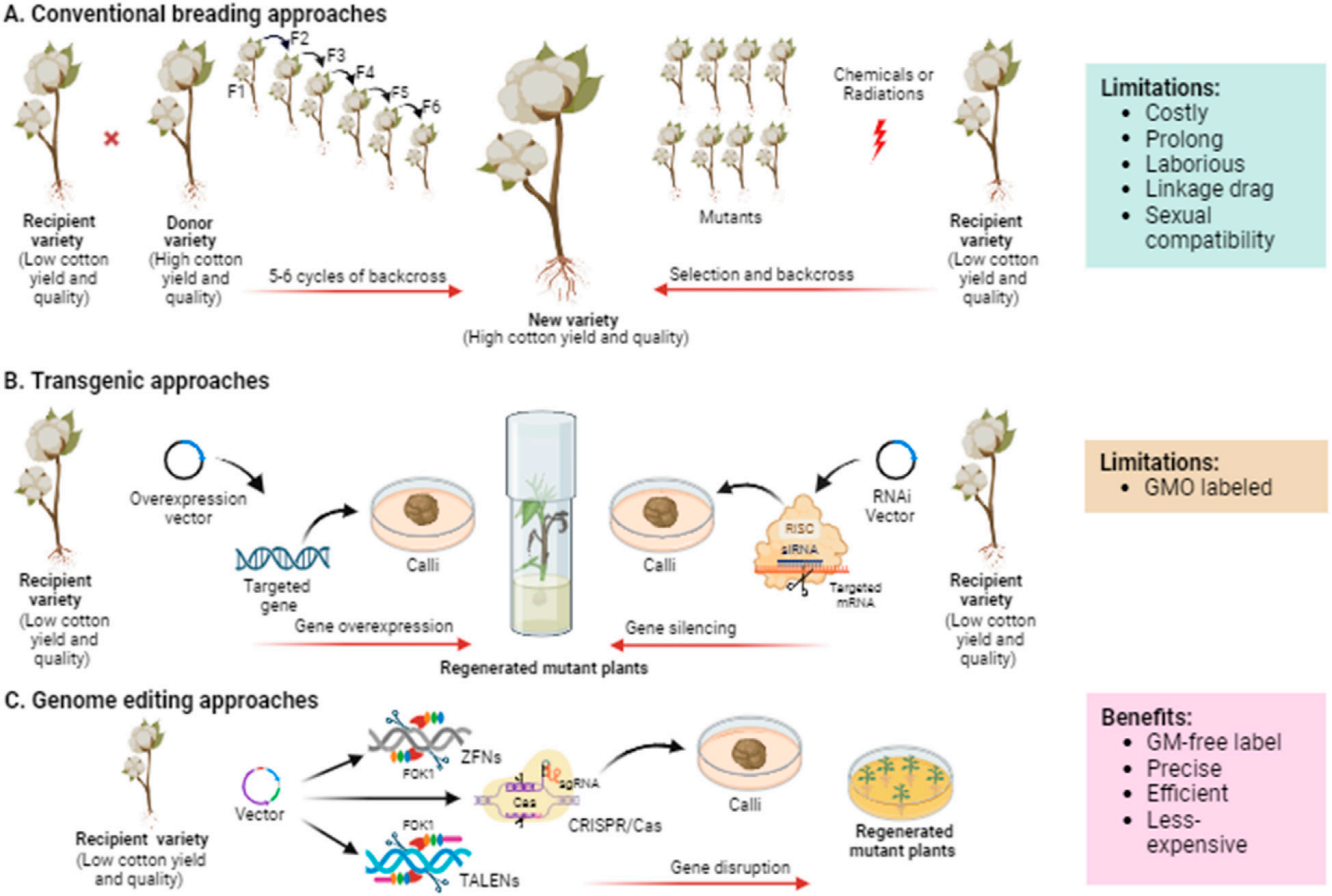
Schematic illustration demonstrating the evolution of breeding technologies from traditional to current genome editing exploited for cotton crop improvement. **(A)** Plant breeding mainly needs the selection of parental genotypes based on phenotype or molecular markers followed by repetitive backcrossing to obtain the progeny with desirable traits. In mutation breeding, the seeds are treated with chemical or physical mutagens to generate mutants, which then undergo rigorous selection to evaluate the desirable phenotype. **(B)** Transgenic approaches are based on introducing a foreign DNA or element into the genome to improve the crop via overexpressing or RNAi-mediated silencing of the targeted gene controlling the trait of interest. **(C)** Genome editing approaches such as ZFNs, TALENs, and CRISPR/Cas are based on targeting and disrupting any specific genes to be improved. ZFNs and TALENs employ FokI endonuclease to cleave DNA double strands, while CRISPR/Cas uses sgRNA for DNA or RNA binding and Cas proteins for DNA or RNA cleavage.

Plant breeding frequently uses “selection” to carefully choose plants with superior traits from a diverse population and breed them multiple generations until these desirable traits become fixed in the selected population (Allard, 2002) (Figure 3A). The Key challenges associated with selective breeding include identifying desirable phenotypes through genetic variation, ensuring sexual compatibility between plants, and linkage drag, which make the approach costly, time-consuming, and labor-intensive.

Marker-assisted selection (MAS) involves the use of various markers to select a trait of interest. These markers can be categorized into three types: morphological, biochemical, and DNA markers. Morphological markers rely on the presence or absence of specific physical traits, while biochemical markers involve the analysis of distinct forms of an enzyme known as allozymes, which are encoded by different alleles at the same locus. DNA markers can be PCR-based, such as Random Amplified Polymorphic DNA (RAPD) (Lu and Myers, 2002), Amplified Fragment Length Polymorphism (AFLP) (Álvarez and Wendel, 2006), or non-PCR-based, such as Simple Sequence Repeats (SSRs) (Li et al., 2018) and Single Nucleotide Polymorphisms (SNPs) (Dilnur et al., 2019), among others. The challenges associated with the selection method can be overcome by utilizing marker-assisted selection (MAS), particularly the DNA-based approach. DNA-based MAS is a cost-effective and time-saving breeding method as it is not influenced by environmental factors and is not dependent on the growth stage of the plants. This makes it highly effective for implementation in early generations of breeding programs. It also enables the selection of precise parental lines for backcrossing, facilitating the introgression of multigenic traits (Frisch and Melchinger, 2005).

### 4.2 Mutation breeding

Mutation breeding involves inducing random mutations in crop plants using mutagens such as Ethyl Methane Sulphonate (EMS) or X-rays (Pathirana, 2011) (Figure 3A). Some of these random mutations may lead to useful traits. Subsequently, mutants with desired traits are identified, self-pollinated, or backcrossed to generate desired progenies. Cotton mutants with improved yield and fiber were developed using gamma rays, EMS, and salicylic acid (Muthusamy and Jayabalan, 2011). Advancements in mutation breeding and functional genomics have facilitated identify genes associated with traits such as plant architecture, leaf colour, and fiber development. In a recent study, Wang G. et al. (2024) developed an EMS mutant library of Asiatic cotton, identifying candidate genes in M1 mutants with different phenotypic variations in traits including, fiber, flower, leaf, and plant architecture through genome-wide mutation analysis. Further, they also confirmed the functions of *GaYUC4* and *GaPDX1* using virus-induced gene silencing, which are candidate genes for bar blade and yellow leaf vein.

For leaf colour-related genes, researchers observed a particular 0.34 Mb hypermutation interval on chromosome D10, containing 31 genes in the F2 population of the Sumian 22 mutant. Among these genes, only the ABCI1 gene showed particularly lower expression in mutants, correlating with decreased levels of chlorophyll-associated compounds. A crucial A to T mutation at −317 bp from the ABCI1 start codon likely hinders its transcription, resulting in the green mutation through impeding chlorophyll synthesis (Gao et al., 2021). For plant architecture-related genes, scientists cloned the Asiatic cotton axillary flowering (*GbAF*) mutant gene as well as the upland cotton cluster branch (cl1) mutant gene, identifying a crucial mutation in which aspartic acid is substituted by asparagine at position 73 in *GbAF*, causing cotton bolls to develop directly on the main stem of the cotton plant. These results indicate that cotton SFT and SP gene orthologs can be used to improve cotton plant architecture (Si et al., 2018).

For fiber-related genes, a short fiber mutant was discovered in the Ghir_A12G008870 gene encoded the tetrapeptide repeat-like superfamily protein in the Ethyl Methane Sulphonate mutant library. VIGS-based silencing of this gene decreased the fiber length in the wild-type cotton line named MD15 (Fang et al., 2020). In addition, a recessive tufted-fuzzless seed mutant was also identified on chromosome D04 having a genome interval of about 411 kb, with 7 genes showing significant differential expression between the tufted-fuzzless seed mutant and wild-type in that region. Researchers discovered a chemically-induced cotton mutant with short fiber, Ligon-lintless-y (liy), modulated through a single recessive locus, which affected many traits such as maturity, fiber length, and plant height (Naoumkina et al., 2021). Furthermore, F-actin polymerization was affected in the mutant *GhACT17D* from Li1 plants due to the substitution of Val for Gly65 on the nucleotide-binding domain of *GhACT17D*. Actin filaments in Li1 fibers indicated decreased filament skewness, greater filament density, faster growth and shrinkage rates, and parallel arrangements compared to the WT control (Cao et al., 2021).

Despite the relative simplicity of the physicochemical mutagenesis tool and the ease of creating mutant populations, the mutagenesis method is not well controlled, and frequently a mutant has more point mutations than the original, which could be the result of multiple point mutations acting in concert to appear the phenotype. In addition, post physicochemical mutagenesis, the plant genome could experience DNA fragment rearrangements or deletions, and urge the transposition of the reverseposon, making it more challenging to characterize functional genes.

## 5 Transgenic approaches

Transgenic approaches have the potential to overcome many of the limitations associated with conventional breeding methods. These approaches allow for the transfer of desired genes into crop plants, regardless of their origin. Transgenic approaches enable the stacking or pyramiding of multiple desired traits in crops. For instance, Bt cotton, a transgenic crop, has rapidly gained acceptance and has been commercially grown in recent years following its introduction (James, 2011). Advancements in functional genomics have facilitated the identification and characterization of a large number of potential genes encoding transcription factors (TFs) that are involved in important traits in cotton. Studies in this field have typically employed two major approaches for gene manipulation: overexpression of native genes and silencing of native genes (Figure 3B).

### 5.1 Gene overexpression

Gene overexpression involves the amplification of desired protein production in plants by utilizing expression vectors that enhance the transcription of the target gene (Chakravarthy et al., 2012). The process is relatively straightforward: genes of interest are cloned into plant expression constructs containing constitutive or tissue-specific promoters based on specific requirements. These cloned genes are then randomly integrated into the plant genome using various available plant transformation methods. Transgenic plants that overexpressed the desired gene are subsequently compared with wild-type plants to assess phenotypic traits associated with the target gene (Wilkins et al., 2000). In their study, Jiang et al. (2012) overexpressed the *GhSusA1* gene, which encodes SUCROSE SYNTHASE, a crucial enzyme for cellulose biosynthesis, playing a pivotal role in secondary cell wall synthesis and fiber cell elongation. Wang et al. (2010) conducted a study in which they overexpressed the gene encoding a fiber-preferential actin-binding protein, *GhPFN2*, resulting in the termination of fiber cell elongation with a short-fiber phenotype. Zhang J. et al. (2017) overexpressed the *GhFIM2* gene encoding an actin-bundling protein, which accelerated fiber growth through boosted actin bundle formation.

### 5.2 RNA interference

RNA Interference is an intrinsic mechanism in plants that regulates gene expression by utilizing small interfering RNAs (siRNAs) to induce post-transcriptional gene silencing (Hannon, 2002). The RNAi process involves two types of small RNAs: siRNAs and microRNAs (miRNAs), which are derived from exogenous or endogenous long double-stranded RNA molecules. These small RNAs guide RNA-induced silencing complexes (RISC) to bind to complementary mRNA sequences, leading to mRNA degradation or translation inhibition, thereby reducing the expression of target proteins. Researchers have developed a powerful RNAi tool called virus-induced gene silencing (VIGS), which allows for rapid and high-throughput functional validation of genes of interest and analysis of their phenotypic effects through transient post-transcriptional gene silencing. Li et al. (2005) used RNAi to repress the *GhACTIN1* gene expression in cotton, which disrupted the actin cytoskeleton network with a significantly decreased fiber elongation while fiber initiation was unaffected. A study reported by Walford et al. (2011), in which the *GhMYB25-like* gene silenced by RNAi, resulted in fiberless cotton seeds. In a study, the silencing of the *GhHOX3* gene expression, which encodes the homeodomain-leucine zipper transcription factor, was achieved by RNAi, resulting in reduced fiber length (Shan et al., 2014). Abdurakhmonov et al. (2016) silenced the phytochrome red/far-red photoreceptor gene (*GhPHYA1*) in cotton through RNAi, which enhanced fiber quality and agronomic traits. The expression of the pigment gland formation gene (GoPGF) encoding a bHLH transcription factor was repressed through VIGS, which results in the emergence of leaves that are either glandless or possess very few glans (Ma et al., 2016). Kumar et al. (2018) developed RNAi cotton lines with silencing of *GhHDA5* gene expression, which encodes HISTONE DEACETYLASE and is involved in fiber initiation.

Despite several studies of crop improvement achieved by transgenic approaches, only some of these transgenic crops have been successfully transferred into usable products for supply and cultivation. This is because most transgenic crops have antibiotic markers and reporter genes for their effective selection. Current biosafety rules and regulations do not permit field trials of transgenic crops having makers and reporters’ genes, and making marker-free and reporter-free transgenic crops is more time-consuming, tedious, and expensive. Additionally, transgenic crops are not being socially accepted as food in some countries.

## 6 Genome editing approaches

Classical plant breeding methods and the adoption of transgenic crops have significantly improved agricultural yield and quality worldwide. However, classical breeding approaches are time-consuming, while the regulatory procedures surrounding transgenic crop availability for food consumption can be complex. Genome editing offers a precise means of manipulating the DNA of cells or organisms by targeting specific DNA sequences and creating double-strand break at desired sites using sequence-specific nucleases. Currently, three main approaches are employed for genome editing, utilizing different types of artificially engineered sequence-specific nucleases: zinc finger nucleases (ZFNs), transcription activator-like effector nucleases (TALENs), and clustered regularly interspaced short palindromic repeats/CRISPR-associated protein (CRISPR/Cas) systems (Carroll, 2014) (Figure 3C).

### 6.1 ZFNs-based approach

The zinc finger nucleases (ZFNs) based approach is an early and widely used site-specific genome editing method. ZFNs are composed of two domains: a zinc finger domain responsible for recognizing specific DNA sequences and a nuclease domain for cleaving DNA (Urnov et al., 2010). These synthetic proteins have been employed as genome-editing tools to introduce various types of mutations, deletions, and insertions in many plant species (Weinthal et al., 2010). Despite the successful studies of ZFNs in plant genome editing, several limitations have hindered their broader application in crop improvement. The main challenges include the complexity and high cost of constructing ZFNs for each specific genomic target, which require extensive knowledge of the target DNA sequence and custom zinc finger protein synthesis. In addition, ZFNs can be toxic and affect the overall health and viability of the plant. Their efficacy can vary, and there’s a risk of off-target mutagenesis due to imprecise protein-DNA interactions (Puchta and Höhn, 2010).

### 6.2 TALENs-based approach

A TALENs-based approach was developed by fusing transcriptional activator-like effector repeats with the FokI endonuclease, aiming to enhance the safety, approachability, and efficiency of genome editing in plants. This system comprises a DNA binding domain derived from Transcription Activator-Like Effectors (TALEs) and a DNA cleavage domain from the FokI endonuclease (Christian et al., 2010). TALENs have been successfully employed in various crops for genome editing and improvement purposes (Khan et al., 2016). Despite TALENs being a significantly improved genome editing tool for crop improvement, they do have certain limitations. These include the necessity of a T nucleotide before the 5′end of the target sequences for selecting TALEN sites, the complexity of designing TALE repeats, the high cost associated with protein engineering, difficulties in delivering these large proteins into plant cells, and their relatively low specificity (Luo et al., 2015).

### 6.3 CRISPR/Cas-based approach

The CRISPR/Cas-based approach has emerged as a high-throughput tool in cutting-edge genomics, with recent studies highlighting its widespread use in genome editing across various plant varieties. This approach offers simplicity, cost-effectiveness, and flexibility compared to ZFNs and TALENs (Zhang et al., 2019). CRISPR/Cas genome editing systems consist of a user-defined 20-nucleotide sequence of a single guide RNA (sgRNA) with a scaffold for attaching the Cas protein, derived from bacteria, which exhibits nuclease activity. To cleave the target sequence, the Cas protein requires a short conserved sequence known as the proto-spacer motif (PAM), which must be located downstream of the target site and cleaved at 3-4 nucleotides after the adjacent PAM sequence (Cong et al., 2013). CRISPR/Cas-based approach provides several advantages over ZFNs and TALENs including RNA-based recognition and cost-effective by eliminating the requirement for protein engineering. CRISPR/Cas allows multiplex editing with multiple gRNAs targeting multiple genes using a single Cas protein. The smaller CRISPR/Cas constructs are easier to deliver into cells, and CRISPR/Cas can recognize methylated DNA, which ZFNs and TALENs cannot (Gaba et al., 2021).

Genome-edited plants are more likely to be accepted by the public than genetically engineered-ones. This is because the integrated transgene such as Cas can be eliminated in subsequent generations by segregation, making these plants non-transgenic, which may not face regulatory scrutiny (Podevin et al., 2012). Unlike mutation breeding, the specific genomic mutation is known in genome-edited plants, and there is no need for backcrossing to eliminate undesired mutations. Unlike transgenic approaches, genome editing through targeted transgene integration by homologous recombination reduces risks such as disrupting gene function or producing toxic proteins. Despite the high costs of ZFNs and TALENs, genome editing provides efficient, precise, and cost-effective crop improvements, enabling gene knockout, modulation of gene expression, and multiple transgenes stacking (Curtin et al., 2012). Hence, genome editing techniques hold great promise as powerful tools for precise genomic modification, enabling researchers to study gene function and develop crops with improved agricultural traits.

## 7 Single-cell RNA sequencing approach

Single-cell RNA sequencing has made it feasible to characterize each transcript present within a single cell. Functional genomics research on plants including cotton has significantly improved with single-cell RNA sequencing, which enables an understanding of gene functions and regulatory networks behind the crucial traits (Peng et al., 2021). With the advent of single-cell RNA sequencing, significant development has been made in the cotton genome and the transcriptome landscape (Wen et al., 2023). Several studies on cotton gene expression have used single-cell RNA sequencing, which has provided important new understandings of various biological processes. Qin et al. (2022) identified core transcription factors such as *MYB25-like* and *HOX3* as crucial for cotton fiber differentiation and growth through single-cell RNA sequencing. By single-cell RNA sequencing, Wang D. et al. (2023) studied rhythmic fiber cell growth, finding that circadian processes, along with small peptide RALF1 and cis-regulatory elements such as CRE and TCPs, play important roles. In a study, Sun et al. (2023) explored gland morphogenesis in *Gossypium bickii*, finding that light and gibberellin promote pigment gland formation, affected by genes like *ERF114*, *ZAT11,* and *NTL9.* Long et al. (2023) also identified transcription factors like *PGF*, *ERF12*, and *MYB14*, as modulators of pigment gland morphogenesis. Lin et al. (2023) identified a hierarchical transcriptional network for terpenoid biosynthesis using single-cell RNA sequencing, with *HSF4a* and *NAC42* directly influencing gene expression in secretory glandular cells. In a recent study, Zhu et al. (2023) compared regenerable and recalcitrant cotton genotypes, identifying genes including *PLT3*, *LOX3*, and *LAX1/2* significant for cell fate reprogramming and plant regeneration. Guo et al. (2024) employed Single-cell RNA sequencing to map the transcriptome during somatic embryogenesis, revealing distinct cell clusters. Recently, Li P. et al. (2024) and Li Y. et al. (2024) studied transcriptome variations in cotton under environmental stress, showing gene expression deviation in particular cell types in anther and root under high temperature and salinity stress. Hence, single-cell RNA sequencing improves cotton functional genomics by offering comprehensive insights into gene functions at the single-cell level. It contributes to a deeper understanding of the genetic and epigenetic mechanisms underlying complex traits in cotton.

## 8 CRISPR/Cas-mediated genome editing in cotton

CRISPR/Cas as a cost-effective, robust, and dominant genome editing tool, has the potential to decrease the time required to develop new cotton varieties and increase sustainable cotton production by editing candidate cotton gene(s). In this section, we discussed the different types of CRISPR/Cas systems that can be used as genome editing tools for cotton improvement and overcoming the challenges in genome editing in cotton.

### 8.1 CRISPR/Cas systems as a genome-editing tool for cotton

**FIGURE 4:**
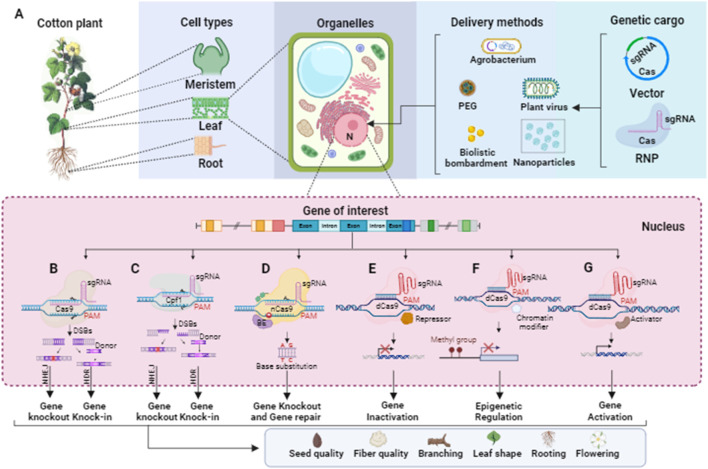
Schematic representation of CRISPR/Cas-based editing tools for targeting the genes related to seed quality, fiber quality, and architecture of the cotton plant. **(A)** DNA or RNA that encode Cas and sgRNAs or CRISPR–Cas–sgRNA ribonucleoprotein (RNP) can be transformed into the nucleus of the meristem, leaf, and root cells of a cotton plant using an appropriate delivery method such as Agrobacterium, plant viruses, polyethylene glycol (PEG), nanoparticles, and biolistic bombardment. **(B)** CRISPR/Cas9 comprises Cas9 endonuclease and a sgRNA complex, and a PAM site (NGG) is present downstream of the targeted DNA sequence. DSBs (blunt-ended) produced by CRISPR/Cas9 fixed by NHEJ or HDR, resulting in gene knock-out or knock-in. **(C)** CRISPR/Cpf1 consists of Cpf1 endonuclease and a sgRNA to bind with targeted DNA which is present upstream of a PAM site (NTT). Similarly, the DSBs (sticky-ended) generated by CRISPR/Cpf1 fix by NHEJ or HDR to gene knock out or knock-in. **(D)** In base editing, a base editor like adenine deaminases or cytidine deaminases is fused with nickase Cas9 (nCas9), which can lead to a base substitution in the targeted DNA sequence for gene knock-out and repair. **(E)** The dead Cas9 (dCas9) fused with a transcriptional repressor to regulate the targeted cotton genes associated with desired traits. **(F)** Epigenome editing at the target genomic site can be executed via Cas9-chromatin modifier fusion protein. **(G)** The dead Cas9 (dCas9) fused with the activator protein can be employed for regulating the expression of the desired genes.

The discovery of CRISPR in the microbial immune system has led biotechnologists to adopt CRISPR and its Cas proteins for RNA-guided genome editing tools in plants. To optimize their utility, several modifications have been introduced. One modification involves the design of a single chimeric guide RNA (sgRNA) controlled by a promoter, replacing the need for expressing two non-coding RNAs (tracrRNA and pre-crRNA). Codon optimization is another modification performed on Cas proteins to ensure appropriate transcription in higher eukaryotic cells. Additionally, these proteins may be fused with nuclear-localized signals to facilitate their transport to the cell nucleus. In engineered CRISPR/Cas systems for gene editing, the gRNA typically consists of a short synthetic RNA that incorporates a trans-activating crRNA (tracrRNA) scaffold for binding with Cas proteins. The gRNA also contains a spacer region with a complementary sequence of twenty nucleotides, targeting the specific site of interest. Cas nucleases possess two nuclease domains, with one cleaving the sense strand and the other cleaving the antisense strand of a targeted gene. This flexibility allows the gRNA to target either strand, guiding the Cas enzyme to make precise cuts at specific sequences within the gene. Moreover, the presence of a protospacer-adjacent motif (PAM) sequence immediately adjacent to the target site is crucial for Cas nuclease activity. The PAM sequence acts as a binding signal for the Cas enzyme, ensuring its specificity. The delivery of CRISPR/Cas reagents into plant cells is mediated by various methods like Agrobacterium, biolistic, polyethylene glycol (PEG), nanoparticle, and plant viruses, as shown in Figure 4A.

#### 8.1.1 Gene knockout

When using CRISPR/Cas9 for gene editing, the system typically cuts the double-stranded DNA at a specific site, resulting in a double-strand break (DSB). The repair of this DSB is primarily carried out through the non-homologous end joining (NHEJ) pathway (as shown in Figure 4B). During NHEJ repair, if one or more nucleotides are added or deleted at the site of the break, it can lead to a frameshift mutation. This frameshift mutation disrupts the reading frame of the targeted protein-coding gene, resulting in the loss of its normal function. This is the basis for quick and precise gene knockout without significant off-target effects. CRISPR/Cas9-based gene knockout is employed for the functional study of a protein-coding gene and for eliminating some undesirable attributes controlled by particular genes in cotton. Gao et al. (2017) reported a rapid and effective methodology for validating the functionality of sgRNAs that target three distinct genes *GhPDS*, *GhCLA1*, and *GhEF1* in cotton, employing both transient and stable transformation. Particularly, they targeted *GhEF1* to check the efficacy of transient transformation-based sgRNA under the control of the AtU3b promoter. Moreover, they accomplished multiple gene targeting in cotton, inducing mutations in *GhPDS* and *GhEF1* at two respective target sites, in which the *GhPDS* and *GhEF1* sgRNAs were under the control of AtU6-29 and AtU3b promoters, respectively. The editing efficiency was high, with 80.6% of transgenic plants exhibiting mutations at the *GhCLA1* target site. Li et al. (2017) successfully knocked out a cotton *GhMYB-25* transcription factor gene on both A and D genomes using CRISPR/Cas9 with 98.8%–100% mutation frequency and no off-targeting. The knock-out line showed a fiber-less phenotype without changing other phenotypic characteristics. Chen et al. (2017) reported genome editing and targeted mutagenesis of the Cloroplastos alterados 1 (*GhCLA1*) and vacuolar H+-pyrophosphatase (*GhVP*) genes in upland cotton (*Gossypium hirsutum* L.) protoplast, employing the CRISPR/Cas9 system. The mutation efficiencies ranged from 47.6% to 81.8% in transgenic cotton lines with no observed off-target mutations. Wang N. et al. (2017) knocked out the cotton *GhARG* gene on both A- and D-genome using the CRISPR/Cas9 system with the editing efficiency ranging from 10% to 98%. The knock-out line exhibited notable development of the lateral root system, enhanced lint yield, more nutrient absorption, and improved tolerance to drought. In a study by Wang Y. et al. (2017), the CRISPR/Cas9 system was utilized to conduct multiple-site genome editing of endogenous gene *GhCLA1*, with the resulting mutated phenotype and genotype transferred to their T1 progenies. The editing efficiency at each target site was 66.7%–100%, without any off-target editing at the potential off-target sites. In another reported study, a *GhALARP* gene, encoding an alanine-rich protein found in fiber cells, was edited via the CRISPR/Cas9 system, with mutation frequency ranging from 71.4% to 100%. Additionally, no off-target mutation event was observed in any predicted sites analyzed (Zhu et al., 2018). Zhang et al. (2018) knocked out the cotton *Gh14-3-3d* gene using the CRISPR/Cas9 system, and homozygous mutated plants showed resistance to *Verticillium dahlia* compared with wild-type. The edited phenotypes were stably passed on T1 generation as well and some homozygous mutants were also achieved. The first report on producing high-oleic acid, nontransgenic mutants in allotetraploid upland cotton through the CRISPR/Cas9 editing system (76% editing efficiency) was recently published by Chen et al. (2020). The mutant cotton lines with a knockout of the microsomal x-6 fatty acid desaturase (*GhFAD2*) gene revealed significant increases in oleic acid along with a decrease in linoleic acid. Lei et al. (2022) developed an effective gene-editing system for rapidly producing cotton mutants via pollen as a transgenic receptor, with minimal off-target effects. They designed tissue-specific vectors to express Cas9 using *GhPLIMP2b* and *GhMYB24* promoters and sgRNAs targeting *GhCLA1*, *GhERA1*, and *GhGGB* genes, which mainly induced base substitutions, with editing efficiencies ranging from 3.29% to 6.45%.

#### 8.1.2 Gene knock-in

In CRISPR/Cas9-based gene knock-in, the CRISPR/Cas9 system is used to target and cut the DNA at a specific position within the genome. Following the DNA cut, a foreign gene or DNA sequence is precisely incorporated into this targeted position through a process called homology-directed repair (HDR) (as shown in Figure 4B). This approach allows for the insertion of a desired gene without causing positional effects. However, CRISPR/Cas9-based gene knock-in is generally more challenging compared to gene knockout, as it often exhibits lower efficiency in genome editing.

#### 8.1.3 Base editing

Genome-wide association studies have provided evidence that single nucleotide changes play a significant role in variations observed in desirable traits in crop plants (Henikoff and Comai, 2003). To address this, the CRISPR/Cas9-mediated base editing tool has emerged as a powerful approach for precisely modifying a single DNA base without the need for a DNA repair template (Komor et al., 2016). In the base editing approach, researchers have modified the Cas9 nuclease into a nickase form called nCas9, which introduces a single-stranded break in the DNA. The nCas9 is then fused with a base conversion enzyme such as cytidine deaminase or adenine deaminase (as shown in Figure 4D). CRISPR/Cas9 base editing has also been employed to disrupt gene function in plants by inducing nonsense mutations with high efficiency compared to the NHEJ process used in CRISPR/Cas9-induced knockouts (Billon et al., 2017). In a study reported by Qin et al. (2019), a novel G. hirsutum-Base Editor 3 (GhBE3) base-editing system has been developed to induce point mutations in the allotetraploid genome of cotton. This system involved fusing a cytidine deaminase with nCas9 and uracil glycosylase inhibitor into a CRISPR/Cas9 vector to target genes *GhCLA* and *GhPEBP*. The editing efficiency of GhBE3 ranged from 26.67% to 57.78% at the target sites. In addition, only <0.1% C>T substitutions were observed in the editing windows of predicted off-target sites, and the edited bases were inherited by T1 progeny. Wang W. et al. (2024) developed highly efficient base editors (GhABE8e) in cotton, which showed 99.9% editing efficiency with no detected off-target mutations. Further, these base editors were utilized to edit both non-coding and coding regions of cotton TERMINAL FLOWER 1 (*GhTFL1*), which are involved in cotton plant architecture. Utilizing 26 targets, they produced a comprehensive allelic population to explore the functional divergence of *GhTFL*1.

#### 8.1.4 Gene regulation

CRISPR/Cas9 system has been harnessed for gene regulation purposes by targeting gene promoters, transcription factors, and enhancers, utilizing either the non-homologous end joining (NHEJ) or homology-directed repair (HDR) processes (Gilbert et al., 2013). The Cas9 endonuclease is commonly employed in CRISPR/Cas9-based gene editing and possesses multiple functional domains, including a PAM-binding domain, as well as RuvC and HNH domains. The RuvC and HNH domains are responsible for cutting the double-stranded DNA, leading to the generation of double-strand breaks (DSBs), which can subsequently be repaired through NHEJ or HDR mechanisms. By deactivating the nuclease activity of the HNH and RuvC domains in Cas9 (resulting in a deactivated Cas9 or dCas9), the binding capability of dCas9 to target sequences remains intact. This property has been exploited in the development of CRISPR/dCas9 interference (CRISPRi) and CRISPR/dCas9 activation (CRISPRa) systems for gene knockdown and gene activation, respectively (Qi et al., 2013). In these systems, dCas9 is fused with either a repressor or an activator, acting as a “gene switch” at the transcriptional level. The binding of dCas9 inhibits the binding of other proteins to the DNA sequence on which dCas9 is already bound, allowing for precise control of gene expression (Gilbert et al., 2013) (as shown in Figures 4E, G).

#### 8.1.5 Epigenetic regulation

Gene expression can be epigenetically regulated through DNA methylation or demethylation at specific nucleotide sites, such as CpG, CHH, and CHG, in plants (Adli, 2018). To target and modulate DNA methylation, the CRISPR/dCas9 system has been modified by fusing the deactivated Cas9 (dCas9) protein with either a methyltransferase or a demethylase, allowing for efficient editing of DNA methylation patterns (as shown in Figure 4F). Papikian et al. (2019) developed a robust and efficient CRISPR/dCas-SunTag system for plants, which incorporates the catalytic domain of the *Nicotiana tabacum* DRM methyltransferase. This system enables precise editing of DNA methylation at specific sites.

#### 8.1.6 CRISPR/Cas12a

CRISPR/Cas9 system has a limitation in target site selection due to its recognition of G-rich protospacer adjacent motifs (PAMs). To overcome this limitation, the CRISPR/Cpf1 system, also known as Cas12a, was developed as an alternative to Cas9. Cpf1 recognizes T-rich PAM sequences (5′-TTN-3′) and generates cohesive-end breaks instead of blunt-end breaks produced by Cas9 (Zetsche et al., 2015) (as shown in Figure 4C). In the CRISPR/Cpf1 system, a single CRISPR RNA (crRNA) of 42 nucleotides is sufficient for target site recognition and cleavage, eliminating the need for tracrRNA used in the Cas9 system. Cpf1 possesses a single nuclease domain that generates a staggered double-strand break (DSB) with a 5′overhang of 4 to 5 nucleotides. This feature makes Cpf1 particularly suitable for homology-directed repair (HDR)-based gene editing, as the ends of the DSBs become predictable. Unlike most Cas nucleases that require PAM sites at the 3′end of the target DNA sequence, Cpf1 requires the PAM site to be positioned at the 5′end. As a result, Cpf1 cleaves the target DNA sequence at the distal end from the PAM, creating the potential for subsequent rounds of cleavage (Zetsche et al., 2015). A LbCpf1 (LbCas12a) plant expression vector with a 23-nucleotide crRNA was used to target the *GhCLA* gene in cotton (*G. hirsutum*). The findings demonstrated an editing efficiency exceeding 80% with no detected off-target effects. The edited traits were stably passed on to the T1 generation with certain homozygous mutants (Li J. et al., 2019). Li et al. (2020) knocked out the GhPGF gene in cotton responsible for pigment gland formation using the CRISPR/LbCpF1 (LbCas12a) system for editing under various temperatures. The results showed that the maximum temperature for CRISPR/Cas12a activity was 34°C with editing efficiencies ranging from 67.6% to 91.5%, and a homozygous gossypol-free non-transgenic line was obtained. A multiplexed crRNA-based Cas12a system has been developed to target the various ORFs of the Cotton leaf curl Multan virus (CLCuMuV) genome at multiple sites simultaneously, which showed an editing efficiency of 21.7%–55.6%. Transgenic *Nicotiana benthamiana* plants harboring multiplex LbCas12a-based construct exhibited a significant decrease in virus accumulation compared to the control plant. This multiplex LbCas12a system has the potential to develop virus resistance in cotton plants against begomoviruses (Ashraf et al., 2023).

#### 8.1.7 Gene stacking

Recombinase technology is an earlier approach, in which recombinases have been used for genetic manipulation including site-specific insertion, deletion, or replacement of a target gene. In a reported study by D’Halluin et al. (2013), a re-engineered meganuclease was developed for the pyramiding of genes through the combined targeted genomic DNA cleavage and homologous recombination-based repair. By integrating up to three genes at a time and a specific genomic site, the gene stacking approach has evolved to be an adaptive strategy for improving traits and preventing varying expression of genes at various regions. Aslam et al. (2022) inserted the Cre and PhiC31-based recombination sites in the cotton genome to develop lines for recombinase-mediated gene stacking. Hence, CRISPR/Cas-based genome editing is more advantageous for gene stacking than recombinase technology due to the preciseness and targeted approach even at the first event of gene insertion in the host genome.

#### 8.1.8 Mutant libraries construction

It is a significant task to critically evaluate the functions of all the sequenced genes in a cotton plant genome. By constructing a genetic library full of mutations, this issue can be resolved. The sgRNA’s 20-bp target-binding site was modified to improve the capability of the CRISPR/Cas9 system to target particular genes. CRISPR/Cas9 is a viable and cost-effective tool for conducting forward genetic analysis and genome-wide mutations. This finding opened the door for the developing CRISPR/Cas9-mediated screening of plant mutant libraries in human cultured cells. Numerous mutant strains were produced when pooled sgRNA libraries were inserted into tomatoes (Jacobs et al., 2017). CRISPR/Cas9-mediated mutant libraries in rice have been created by a research group, which has produced a significant number of gene-disrupted mutations by transforming sgRNA libraries (Meng et al., 2017). In cotton, Ramadan et al. (2021) established a CRISPR/Cas9 system-mediated pooled-sgRNAs method to target multiple genes associated with male sterility. A population of mutants was produced, which would be useful to find the key genes that may improve fertility. These findings collectively showed that constructing mutant libraries with CRISPR/Cas systems ensures speedy functional characterization of identified genes, paving the way for future improvement in the cotton genome.

### 8.2 Challenges associated with genome editing in cotton

**FIGURE 5:**
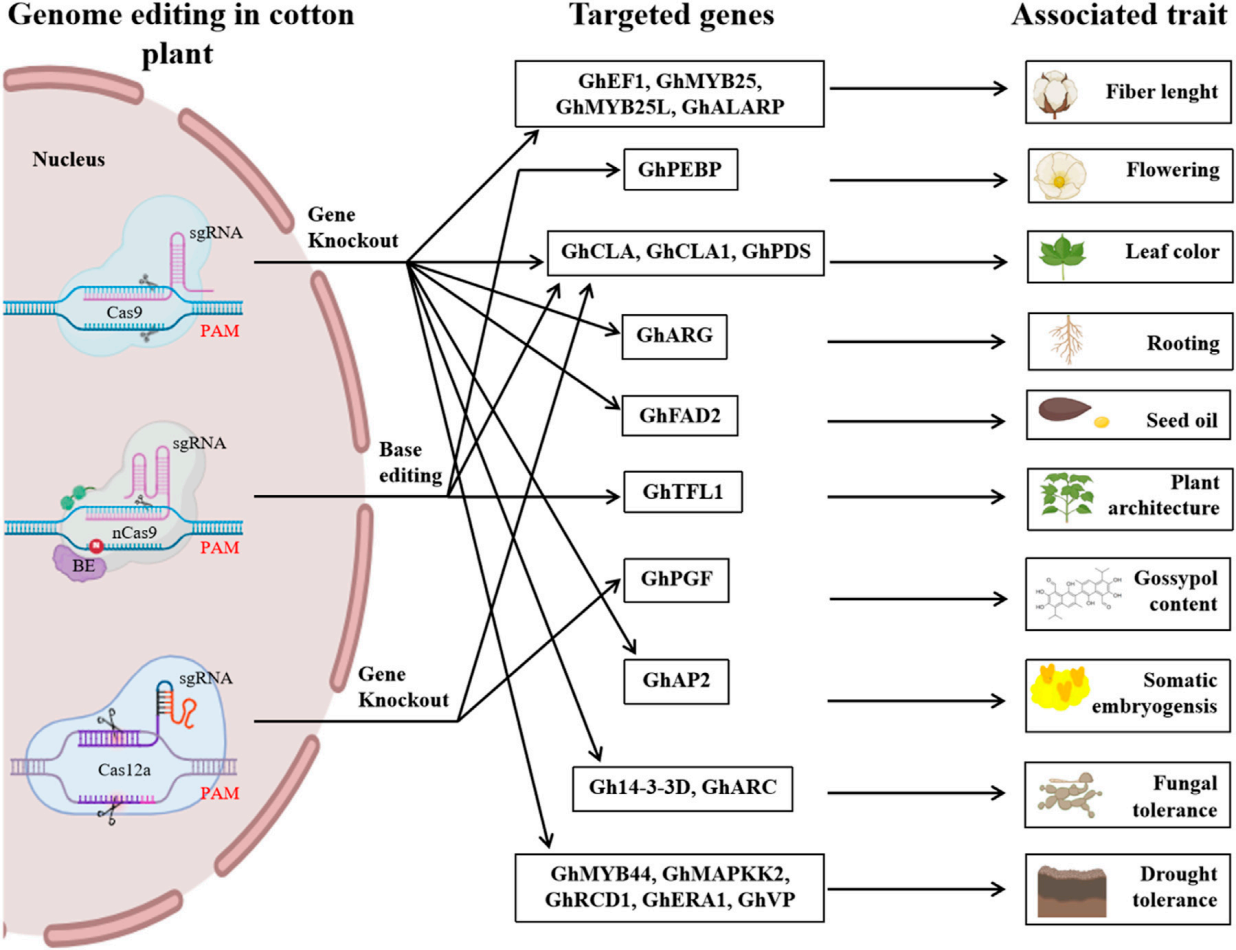
An illustrated overview of different genome editing studies in cotton that were previously reported. It highlights the CRISPR/Cas variants such as Cas9, nCas9, and Cas12a and targeted genes and their associated traits reported in previous studies regarding CRISPR-mediated genome editing in cotton. It underscores the key potential of CRISPR-mediated genome editing to advance cotton breeding and improve traits.

CRISPR/Cas-mediated genome editing has increased biotic and abiotic stress tolerance, yield, and end-use quality of cotton crops, as depicted in Figure 5. However, some challenges remain for genome editing in tetraploid cotton, which we discussed in this section.

#### 8.2.1 Requirement of explants with regenerative potential

The main challenge in generating CRISPR-edited cotton lines is the requirement for explants with regenerative potential. The recalcitrant to Agrobacterium infection and regeneration via somatic embryogenesis are the key bottlenecks in genome editing of elite cotton cultivars. Only these cotton genotypes Coker312, ZM24, JIN668, and YZ-1 have been transformed via Agrobacterium-mediated transformation at very low transformation efficiency. In a study reported by Chen et al. (2017), they delivered the CRISPR/Cas9 system into the tetraploid cotton genome by the vacuum infiltration-assisted Agrobacterium-mediated transformation using the shoot apexes as ex-plant, resulting in obtained mutation frequencies ranging from 47.6% to 81.8%. In another study, Ge et al. (2023) established an efficient and genotype-independent shoot apical meristem cell-mediated transformation (SAMT) system for different recalcitrant cotton genotypes that bypasses somatic embryogenesis. Using this SAMT, they produced edited elite cotton genotypes with inheritable targeted editing in genes (GhRCD1 and GhPGF) through the CRISPR/cas9 system. This study paves a new way for producing gene-edited elite cotton varieties, which are recalcitrant to genetic transformation and regeneration.

Furthermore, several plant viruses like tobacco mosaic virus (TMV), tobacco rattle virus (TRV), and potato virus X have been genetically modified to be used as vectors in VIGS for functional genomics (Lange et al., 2013) and in delivering the ZFNS and TALENs for generating gene knockouts in both monocots and dicots (Cody and Scholthof, 2019). Now, the heritable, efficient, and transgene-free CRISPR-mediated gene editing is also achieved in germline cells of plants using the viral vectors, which are delivered into plants through Agrobacterium. The replicating viral vectors employed for CRISPR/Cas9 delivery can't carry the Cas9 gene due to its considerably larger size. Therefore, the Pea early-browning virus and Tobacco rattle virus have been reported to deliver small gRNAs into genetically modified plants expressing the Cas9 gene for CRISPR/Cas9-mediated gene editing (Hu et al., 2019). Sonchus Yellow Net Rhabdovirus, an RNA virus with higher cargo capacity, was engineered to deliver both the Cas9 gene and small guided RNA into the plant cell for transgene-free In planta CRISPR editing (Ma et al., 2020). Most of these viral vectors can't target the meristematic cells to induce mutations and give the heritable edits from seeds. To overcome this issue, the gRNA has been fused with the endogenous mobile RNA encoded by Flowering Locus T to increase mobility and ease systemic spread within plants to reach the meristematic cells (Ellison et al., 2020). Lei et al. (2022) developed a cotton leaf crumple virus (CLCrV)-mediated VIGE system in cotton using Cas9-overexpressing cotton lines as the receptor. With the use of this VIGE system, it's feasible to simultaneously double-mutate the *GhCLA1* and *GhPDS* genes in addition to knocking out the *GhMAPKKK2*, *GhCLA1*, and *GhPDS* genes on both A and D genomes. It showed high gene editing specificity with mutation efficiencies ranging from 8.02%–52.68%. Additionally, they fused the gRNA with FT mRNA for heritable targeted editing of the *GhCLA1* and *GhPDS* genes in tetraploid cotton, which exhibited editing efficiency of 23.98%–55.43% but heritable mutant progeny was not detected. In another study reported by Gao et al. (2021), a convenient and robust genome editing strategy was developed in cotton through engineered CLCrV-mediated sgRNA delivery, in which the *GhMYB25L* gene (involved in regulating cotton fiber initiation) targeted using this strategy for mutation in stably transformed Cas9-expressing cotton lines. Further, gRNA was expressed and delivered in non-regenerative cotton variety via the grafting method, resulting in each Cas9-expressing cotton plant producing many Cas9-sgRNA cotton plants. The Hi-tom sequencing results of scions from 72 grafted plants showed that every single plant has an editing efficiency of up to 62.15% after 3 weeks of grafting. Hence, CLCrV-mediated genome editing with grafting precludes the requirement for lengthy tissue culture and laborious transformation procedures in cotton.

#### 8.2.2 Requirement of specific protospacer adjacent motifs (PAMs)

The requirement of specific protospacer adjacent motifs (PAMs) has limited the range of DNA sequences that can be targeted for gene editing using the CRISPR/Cas system in cotton. However, Walton et al. (2020) have recently developed modified versions of the SpCas9 nuclease, namely SpG and SpRY, through structure-guided engineering. These modifications relax the PAM requirements, allowing for the targeting of a broader range of DNA sequences for CRISPR/Cas-mediated gene and base editing. Importantly, these modifications do not compromise the Cas nuclease activities. In a study reported by Wang G. et al. (2024), the GhABE7.10-dCpf1 and GhABE8e-dCpf1 vectors were devised and evaluated to expand the PAM sites in the cotton genome, which recognized TTTV PAM sequences. In the GhABE7.10-dCpf1 vector, dCpf1 (deactivated Cpf1) fused with adenine deaminase TadA7.10, which showed editing efficiency ranging from 0.2% to 0.5%, while in the GhABE8e-dCpf1 vector, dCpf1 fused with TadA8e, which showed editing efficiency of 1.5%. The lower editing efficiency than the GhABE8e-Cas9n system may be due to the less compatibility of TadA7.10 and TadA8e to Cpf1.

#### 8.2.3 Low multiallelic editing efficiency

The low editing efficacy is a significant challenge in tetraploid cotton that sometimes needs simultaneous editing of several homoeologous gene copies to obtain the desired phenotype. The gRNA activity and efficiency could be assessed *in vivo* using protoplasts or transient co-expression assays before stable transformation as the creation of transgenic cotton lines usually takes several months. Using the transient expression approach in cotton cotyledons for high-throughput validation of gRNA, Gao et al. (2017) obtained a mutation frequency of above 80% following stable transformation. Although a single gRNA could generate frameshift mutations, using multiple gRNAs to target a single gene could result in significant deletions across target sites and enhance the chance of producing multiallelic loss-of-function mutations. Additionally, high Cas and gRNA transcript levels could boost the effectiveness of gene editing. Thus, the promoters regulating the expression of gene editing reagents have a major effect on obtaining high mutation efficiencies. Long et al. (2023) demonstrated that the editing efficiency of the cotton CRISPR/Cas9 system was improved 4–6 times when gRNA is driven by endogenous GhU6 promoter instead of Arabidopsis AtU6-29 promoter. To enable highly efficient genome editing in tetraploid cotton, Zhang et al. (2018) employed a modified CRISPR vector that comprised the native promoter GhU6.9. Sequencing showed mutagenesis efficiencies ranging from 66.7% to 100% at four target regions. Chen et al. (2020) used the same vector system in a follow-up study and found that 76% of the recovered transgenic plants had mutations in cotton *GhFAD2* homologs. This vector was also employed by Ramadan et al. (2021) to improve their potent pooled-gRNA assembly approach. Li et al. (2022) developed the two novel genome editing vectors named pBeYDV-Cas9-KO (engineered bean yellow dwarf virus) and pRGEB32-35S (Cas9 driven by CAMV35S promoter) and tested them by targeting the *GhCLA1* gene in cotton. Further, they also compared them with the ordinary CRISPR/Cas9 method, which showed that both new genome editing vectors had great efficiency (73.3% and 51.2% by pBeYDV-Cas9-KO and pRGEB32-35S respectively) with no off-target effects. In cotton, Cas expression is mostly driven under the Cauliflower mosaic virus 35S promoter (CaMV 35S), But rice Ubiquitin (OsUbi) promoters are also reported (Table 4). In addition to constitutive expression, Cas can also be expressed in particular cell types. In cotton, the GhPLIMP2b and GhMYB24 promoters were used to induce Cas9 expression in pollen (Lei et al., 2022).

**TABLE 4:** A list of reported studies of the CRISPR/Cas-mediated genome editing in cotton.

Targeted gene	Cas	Cas promoter	gRNA promoter	CRISPR reagents delivered by	Study objective	References
*GhEF1, GhCLA1, GhPDS*	Cas9	*2x35S*	*AtU6, AtU3b*	Agrobacterium^a^ ^,^ ^b^	KO; Loss of function mutation	Gao et al. (2017)
*GhMYB25-A, GhMYB25-D*	Cas9	*2x35S*	*AtU6*	Agrobacterium^b^	KO; Targeted editing of fiber quality associated gene	Li et al. (2017)
*GhCLA1, GhVP*	Cas9	*CAMV35S*	*AtU6*	Agrobacterium^c^	KO; Targeted gene editing in cotton protoplast and shoot apexes	Chen et al. (2017)
*GhARG*	Cas9	*CAMV35S*	*NtU6*	Agrobacterium^b^	KO; Improve lateral root formation	Wang et al. (2017b)
*GhCLA1*	Cas9	*OsUbi*	*GhU6*	Agrobacterium^b^	KO; Loss of function mutation	Wang et al. (2017b)
*GhALARP-A, GhALARP-D*	Cas9	*CAMV35S*	*AtU6*	Agrobacterium^b^	KO; Editing of gene expressed in fiber	Zhu et al. (2018)
*Gh14-3-3D*	Cas9	*CAMV35S*	*AtU3b*	Agrobacterium^b^	KO; Tolerance against *V. dahliae*	Zhang et al. (2018)
*GhPDS*	Cas9	*2x35S*	*AtU6, GhU6*	Agrobacterium^a^	KO; Check the efficiency of gRNA promoters in cotton	Long et al. (2023)
*GhAP2, GhMYB44, GhARC*	Cas9	*OsUbi*	*GhU6*	Agrobacterium^b^	KO; Analysis of off-target activity of CRISPR/Cas9 in cotton	Li et al. (2019a)
*GhCLA1*	Cas12a (cpf1)	*OsUbi*	*GhU6*	Agrobacterium^b^	KO; Targeted gene mutation	Li et al. (2019b)
*GhPGF*	Cas12a (cpf1)	*OsUbi*	*GhU6*	Agrobacterium^b^	KO; Gossypol free cotton	Li et al. (2020)
*GhCLA, GhPEBP*	nCas9	*OsUbi*	*GhU6*	Agrobacterium^b^	BE; Check the efficiency of base editing in cotton	Qin et al. (2019)
*GhFAD2-A, GhFAD2-D*	Cas9	*OsUbi*	*GhU6*	Agrobacterium^b^	KO; Increase oleic acid content	Chen et al. (2020)
*GhCLA1, GhERA1, GhGGB*	Cas9	*GhPLIMP2b, GhMYB24*	*GbU6*	Agrobacterium^c^	KO; Tissue-specific editing in pollen	Lei et al. (2020)
*GhCLA1*	Cas9	*CAMV35S*	*GhU6*	Agrobacterium^b^	KO; Check the efficiency of the geminivirus-mediated gene editing vector in cotton	Li et al. (2022)
*GhMAPKKK2* *GhCLA1, GhPDS*	Cas9	*CAMV35S*	*AtU6*	Cotton leaf crumple virus^a^	KO; Establish a VIGE system based on CLCrV in cotton	Lei et al. (2022)
*GhRCD1, GhPGF*	Cas9	*CAMV35S*	*AtU6*	Agrobacterium^d^	KO; Study the feasibility of SAMT for genome editing in cotton	Ge et al. (2023)
*GhMYB25L*	Cas9	*CAMV35S*	*GhU6*	Cotton leaf crumple virus^a^	KO; Establish a CLCrV-mediated CRISPR/Cas9 and grafting system in cotton	Guo et al. (2023)
*GhTFL1*	nCas9	*OsUbi*	*GhU6*	Agrobacterium^b^	BE; Base editing of plant architecture-related gene	Wang et al. (2024b)

^a^ Agroinfiltration method, Ex-plants; cotyledenary leaves.

^b^ Dip method, Ex-plants; hypocotyls.

^c^ Vacuum infiltration method, Ex-plants; shoot apical meristem and pollen.

^d^ Sonication method, Ex-plants; shoot apical meristem.

KO: Gene knockout.

BE: Base editing.

#### 8.2.4 High off-target effects

The length of gRNA is vital to determine the off-target site within the host genome. The gRNA sequence and the occurrence of a PAM downstream to the gRNA sequence in the genome firmly control the targeting specificity of Cas9, off-target cleavage activity may still take place on DNA sequences with even 3–5 bp mismatches in the gRNA sequence (Kang et al., 2022). In the case of CRISPR/Cas9 systems, the common cause of off-target cleavage activity is the gRNA’s recognition of completely or partially complementary genomic sites (Li et al., 2018). Different CRISPR/Cas variants such as HF-Cas9, eCas9, and HypaCas9 are also present with varying PAM requirements, which can be used to increase on-targeting and reduce off-target. Several approaches have been suggested to reduce off-target effects, including altering the half-time of Cas9 or reducing the amount and duration of functioning Cas9 protein in cells through selective delivery (Hajiahmadi et al., 2019). Furthermore, genome-wide off-targeting analysis can also be performed before the stable transformation of the CRISPR/Cas tool in cotton. Li et al. (2018) demonstrated a whole genome sequencing approach to identify the on and off-target mutation in edited cotton plants. Moreover, the inherent genetic mutation of wild-type can also produce new off-target sites and demolish PAMS, which signified that gRNA is designed very carefully to reduce off-target effect (Li et al., 2018).

#### 8.2.5 Assessment of multiallelic editing

Targeted editing in tetraploid cotton can result in different genotypes such as knockouts in one or more copies or all alleles. Due to this, the molecular characterization of mutations in cotton genomes can also be onerous. The medium-throughput techniques include polymerase chain reaction (PCR) to amplify the genomic target sequence covering gRNA and gel electrophoresis for visualization of longer or shorter indels in the targeted genomic site, accordingly. These techniques reduced the expenses and labor related to genotyping tetraploid cotton plants for screening potential mutants generated by CRISPR/Cas-mediated genome editing. Cleaved amplified polymorphic sequences (CAPS/PCR-RE) assays, T7 endonuclease 1 assay (TE71), single-strand conformation polymorphism (SSCP) method, and high-resolution melting (HRM) curve analysis have been used for the identification of mutations. However, the number and nucleotide sequence of gRNAs delivered determines which screening method should be employed (May et al., 2023). The most often employed technologies have been Illumina sequencing with high-throughput and Sanger sequencing for assessing the type and frequency of mutations in tetraploid cotton. Sanger sequencing offers a longer read length, that evaluates editing at multiple gRNA sites within a single allele and differentiates allelic variations more precisely through identifying allele-specific SNPs. In several reported studies, Sanger sequencing of multiple clones has been also used to quantify the number of co-edited alleles in a cotton line. This approach is useful for assessing either simple or chimeric mutations but it is costly and time-consuming. To avoid multiclone sequencing, various computational tools including Tracking of Indels by Decomposition (TIDE), HI-TOM, Inference of CRISPR Edits (ICE), Cas-analyzer, CRISP-ID, and Deconvolution of Complex DNA Repair (DECODR) greatly facilitates the quantitative evaluation of the extent and type of targeted edits utilizing Sanger sequencing data (May et al., 2023). In addition, third-generation long-read sequencing approaches such as Single Molecule Real-Time (SMRT) and Nanopore can be also used to quantify the number of mutated alleles in tetraploid cotton (May et al., 2023).

Furthermore, commercializing genome-edited cotton is also challenged by stringent and variable regulatory frameworks, technical issues such as off-target effects and trait complexity, and complex intellectual property landscapes. Mixed consumer perspectives and slow industry adoption restrict market acceptance. High development costs, uncertain returns on investment, limited funding, and the difficulty of conducting extensive field trials further complicate progress. Despite these obstacles, potential advantages including improved fiber and seed quality or yield make genome-edited cotton a promising study field, with future feasibility likely as regulations evolve and technical advances are made.

## 9 Key target genes for genome editing in cotton

**FIGURE 6:**
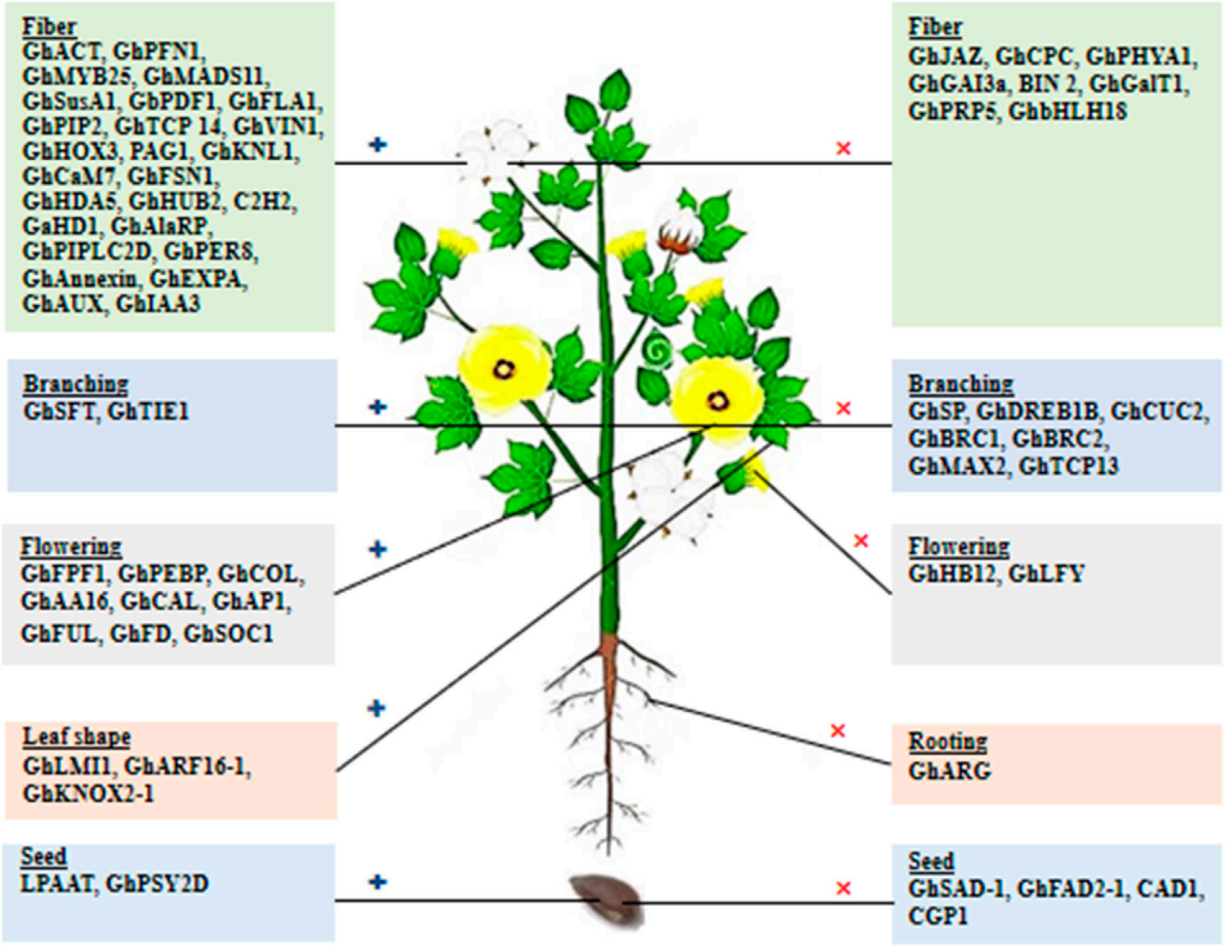
Overview of key target genes for genome editing in cotton. The “+” sign for overexpressing and the “×” sign for knockout or knockdown of these genes by using the CRISPR/Cas-mediated gene editing tool could improve the associated trait.

Whole genome sequencing and multi-omic analysis have been conducted to identify key genes associated with agriculturally important traits, such as fiber and seed quality, as well as plant architecture in cotton. These genes represent potential targets for advanced CRISPR/Cas-mediated genome editing tools to enhance these specific traits (Figure 6). The identified genes can be categorized into two types: structural genes, which primarily consist of protein-coding genes directly controlling the distinctive traits of the crop. Therefore, CRISPR/Cas-mediated genome editing is an excellent choice for improving traits governed by structural genes. However, it's important to note that traits are not solely regulated by these structural genes in many plants. Numerous regulatory genes, including transcription factors and non-coding RNAs, are also involved in the underlying mechanisms. Therefore, targeting transcription factor genes using CRISPR/Cas tools holds promise for enhancing agriculturally important traits in cotton, as these transcription factors may regulate multiple structural genes associated with these traits.

Several transcription factor genes in cotton have been discussed in the previous section as potential targets for CRISPR/Cas genome editing to improve various traits associated with cotton fiber, including yield, length, and strength. These genes include *GhFLA1, GhACT1*, *GhPFN1*, *GhMYB25*, *GhMADS11*, *GhSusA1*, *GhPIP2*, *GhTCP14*, *GhHOX3*, *PAG1*, *GhKNL1*, *GhCaM7*, *GhFSN1*, *GhHUB2*, *GaHD1*, *GhAlaRP*, *GhPIPLC2D*, *GhPER8*, *GhAnnexin*, *GhEXPA*, *GhAUX*, *GhIAA3*, *GbPDF1*, *GhVIN1*, *GhHDA5*, and *C2H2*. These genes serve as positive regulators in the fiber initiation and elongation processes and can be overexpressed by utilizing the CRISPRa tool, which involves the binding of activators (Table 1; Figure 6). Additionally, genes such as *GhJAZ2, GhGAI3a*, *GhCPC*, *GhPHYA1*, *BIN2*, *GhGalT1*, *GhPRP5*, and *GhbHLH18* act as negative regulators in the fiber initiation and elongation processes. These genes can be targeted for knockout or silencing using CRISPR/Cas9 or Cpf1 tools (Table 1; Figure 6).

Several genes have been identified as potential targets for improving cottonseed quality through the use of CRISPR/Cas tools (Table 2; Figure 6). The CGP1 and Δ-CADINENE SYNTHASE (*CAD1*) genes, which are involved in gossypol biosynthesis, can be effectively knocked down using the CRISPRi tool by targeting the seed-specific promoter. This approach allows for the reduction of gene expression without affecting the gossypol levels in other plant tissues. The knockout of STEAROYL-ACYL-CARRIER PROTEIN Δ9-DESATURASE (*GhSAD-1*) and oleoylphosphatidylcholine ω6-DESATURASE (*GhFAD2-1*) genes, which are involved in fatty acid desaturation, through CRISPR/Cas9 or Cpf1-mediated approaches, can enhance the oleic acid content in cottonseed oil. Overexpression of the LYSOPHOSPHATIDIC ACID ACYLTRANSFERASE (*LPAAT*) gene using CRISPRa can increase the oil content in cottonseed, as this gene plays a crucial role in cottonseed oil biosynthesis. Cottonseed can be biofortified using the CRISPRa tool by targeting the PHYTOENE SYNTHASE (*GhPSY2D*) gene, resulting in enhanced Pro-vitamin A content. This biofortification strategy holds promise for addressing vitamin A deficiency on a global scale.

The previously reported genes associated with cotton plant architecture, which regulate flowering, branching in reproductive and vegetative growth, rooting pattern, and leaf shape, represent potential targets for CRISPR/Cas-based editing. These gene modifications aim to improve cotton plant architecture and increase productivity (Table 3; Figure 6). Roots play a crucial role in the plant’s defense system against belowground biotic stresses and serve as sensors for water, nutrients, and environmental conditions. The ARGINASE (*GhARG*) gene can be targeted for knockout using CRISPR/Cas9 or Cpf1 tools to enhance lateral root formation in cotton. This genetic modification can lead to an increase in total root surface area, effectively stimulating both vegetative and generative growth of the entire cotton plant. Ultimately, this improvement can result in increased fiber productivity, especially under drought and low nutrient conditions.

In plants, the leaf is a vital organ responsible for generating food through photosynthesis. Leaf shape plays a crucial role in sunlight interception. Therefore, the overexpression of genes such as LATE MERISTEM IDENTITY1 (*GhLMI1*)-like, AUXIN RESPONSE FACTOR (*GhARF16-1*), and *GhKNOX2-1* genes using CRISPRa tools can improve leaf shape in cotton. This enhancement leads to increased light interception in the leaves, ultimately improving crop productivity. Several reported transcription factor genes involved in branching regulation represent potential targets for CRISPR/Cas-based genome editing to enhance crop management and yield in cotton (Table 3; Figure 6). By using the CRISPRa tool, the expression of *GhSFT*, *GhSP*, and *GhTIE1* genes can be increased, promoting indeterminate and determinate growth by modulating monopodial and sympodial branching patterns. Additionally, CRISPR/Cas9 or Cas12a-mediated knockout of *GhDREB1B*, *GhCUC2*, *GhBRC1*, *GhBRC2*, *GhMAX2*, and *GhTCP13* genes could induce monopodial and sympodial branching in cotton.

Several reported transcription factor genes involved in flower organ development and flowering time in cotton represent potential targets for editing through CRISPR/Cas tools to induce early maturity (Table 3; Figure 6). The overexpression of genes such as *GhFPF1*, *GhPEBP*, *GhCOL*, *GhAA16*, *GhCAL*, *GhAP1*, *GhFUL*, *GhFD*, and *GhSOC1* using the CRISPRa tool, along with the knockout of *GhHB12* and *GhLFY* genes through CRISPR/Cas9 or Cas12a-mediated approaches, can effectively promote the early transition from generative to reproductive growth (early flowering) in cotton. Through CRISPR/Cas-based knockout of negative regulator genes and overexpression of positive regulator genes associated with output traits, significant improvements in the phenotype of cotton plants can be achieved, facilitating desired trait enhancements.

## 10 Future prospective

Despite conventional breeding approaches yielding improved traits in the cotton crop, their lengthy breeding cycles, limited fidelity in hybridization, high level of heterozygosity, and infrequent occurrence of desirable mutations require substantial resources for the development of new cotton varieties. Transgenic approaches have been utilized for cotton improvement due to their ability to overcome compatibility barriers between cotton species. However, these approaches face challenges such as public acceptance, high costs, and time-consuming processes due to strict biosafety regulations. The emergence of CRISPR/Cas tools now offers a promising solution, enabling researchers to generate DNA-free edited crop plants that may be more readily accepted by the public compared to transgenic methods. The simplicity, versatility, and robustness of CRISPR/Cas tools have addressed many drawbacks associated with genome editing, leading to renewed approaches for enhancing cotton crop improvement in terms of precision, efficiency, and time savings. These tools facilitate gene knockout, knock-in, replacement, base-editing, fine-tuning of gene regulation, and epigenetic modifications. However, further research is required to fully understand the potential of CRISPR tools in cotton. By leveraging CRISPR/Cas technology, it becomes possible to enhance agronomic traits, significantly improving total cotton productivity and quality. To tackle the complexity of agronomic traits, multiplex genome editing allows for the efficient stacking of multiple desirable traits in cotton plants. Ultimately, CRISPR/Cas-based genome editing in cotton has the potential to increase food, feed, fuel, and fiber production sustainably, thereby contributing to global food security in the future.
